# Microarray analysis of androgen-regulated gene expression in testis: the use of the androgen-binding protein (ABP)-transgenic mouse as a model

**DOI:** 10.1186/1477-7827-3-70

**Published:** 2005-12-09

**Authors:** Peter Petrusz, Durairaj A Jeyaraj, Gail Grossman

**Affiliations:** 1 Department of Cell and Developmental Biology and Laboratories for Reproductive Biology, The University of North Carolina at Chapel Hill, School of Medicine, Chapel Hill, NC 27599, USA

## Abstract

**Background:**

Spermatogenesis is an androgen-dependent process, yet the molecular mechanisms of androgens' actions in testis are poorly understood. Transgenic mice overexpressing rat androgen-binding protein (ABP) in their testes have reduced levels of intratesticular androgens and, as a result, show a progressive impairment of spermatogenesis. We used this model to characterize changes in global gene expression in testis in response to reduced bioavailability of androgens.

**Methods:**

Total RNA was extracted from testes of 30-day old transgenic and wild-type control mice, converted to cRNA, labeled with biotin, and hybridized to oligonucleotide microarrays. Microarray results were confirmed by real-time reverse transcription polymerase chain reaction.

**Results:**

Three-hundred-eighty-one genes (3.05% of all transcripts represented on the chips) were up-regulated and 198 genes (1.59%) were down-regulated by at least a factor of 2 in the androgen-deficient animals compared to controls. Genes encoding membrane proteins, intracellular signaling molecules, enzymes, proteins participating in the immune response, and those involved in cytoskeleton organization were significantly overrepresented in the up-regulated group. Among the down-regulated transcripts, those coding for extracellular proteins were overrepresented most dramatically, followed by those related to proteolysis, cell adhesion, immune response, and growth factor, cytokine, and ion channel activities. Transcripts with the greatest potential impact on cellular activities included several transcription factors, intracellular signal transducers, secreted signaling molecules and enzymes, and various cell surface molecules. Major nodes in the up-regulated network were IL-6, AGT, MYC, and A2M, those in the down-regulated network were IL-2, -4, and -10, MAPK8, SOCS1, and CREB1.

**Conclusion:**

Microarray analysis followed by gene ontology profiling and connectivity analysis identified several functional groups of genes and individual genes responding to sustained reduction of androgen levels in the mouse testis. These include genes whose products function as transcription factors, cell surface molecules including ion channels, extra- and intracellular signaling molecules, and secreted enzymes with the potential of regulating cell-to-cell attachment. The transcription factors CREB1 (down-regulated) and MYC (up-regulated) may mediate the most important initial phases of the testicular response to reduced levels of androgens. These results suggest specific avenues for further research that will lead to a better understanding of how androgens regulate spermatogenesis.

## Background

Spermatogenesis is an androgen-dependent process, yet the molecular mechanisms of androgens' actions are poorly understood. Both testosterone (T) and its active metabolite dihydrotestosterone (DHT) bind to and activate the androgen receptor (AR), a member of the family of ligand-activated transcription factors, coded for by a single-copy gene on the X chromosome [1]. Although AR in the testis is present in multiple cellular compartments, including Sertoli cells (SC), it is not present in germ cells [2]. It has long been assumed that the actions of androgens on spermatogenesis are mediated largely by SC [3]. Recent studies have demonstrated that SC-selective knockout of AR results in spermatogenetic arrest in meiosis [4-6], confirming that activation of AR in SC is an absolute requirement for androgen maintenance of spermatogenesis.

Transgenic mice overexpressing rat androgen-binding protein (ABP) in their testes [7] show progressive abnormalities of spermatogenesis leading eventually to infertility [8]. Detailed analyses of the dynamics of germ cell proliferation and apoptosis in such animals indicated that most if not all of the testicular abnormalities may be explained by reduced bioavailability of androgens in the testes [9,10]. Direct measurement of testicular total and free T concentrations, at least up to 60 days of age, support this hypothesis [11].

The intratubular compartment in the testes of ABP-transgenic mice is characterized not only by lower than normal concentrations of bioavailable androgens but also by higher than normal concentrations of ABP [8,11]. Although germ cells have been shown to possess receptors for ABP and germ cells actually internalize ABP in both normal and transgenic mice [9,12,13], there is no evidence to date to indicate that ABP may have a direct, deleterious effect on germ cells in normal or transgenic mice. Thus, we reasoned that the ABP-transgenic mice may be used as a model for elucidating the effects of sustained androgen deficiency on gene expression in the testis, offering new insights into the molecular mechanisms of the control of spermatogenesis by androgens. In order to compare testes with similar cellular composition, we selected 30-day old mice for these studies.

## Materials and methods

### Animals

Mice were housed in the animal facility of the University of North Carolina at Chapel Hill under controlled conditions. Food and water were available *ad libitum*. The Institutional Animal Care and Use Committee approved the protocols used in this study. Transgenic mice overexpressing rat ABP in their testes were developed [7] and were propagated as described previously [8,9]. Thirty days old control (wild type, WT, n = 3) and homozygous transgenic (TG, n = 3) male mice were used for these studies. The animals were sacrificed by rapid decapitation after a brief exposure to ether.

### Chemicals

All chemicals used in these studies were obtained from Sigma Chemical Co., St. Louis, MO, unless stated otherwise.

### Target RNA preparation and oligonucleotide array expression analysis

Testes were quickly removed, weighed, and placed in ice-cold TRIZOL (Invitrogen, Carlsbad, CA) solution until further processing. The two testes from each animal were combined and total cellular RNA was isolated using the TRIZOL reagent and purified using RNeasy minicolumns (Qiagen, Valencia, CA) according to the manufacturers' instructions. The yield was 150–200 μg of RNA per animal. The remainder of the procedure was carried out in UNC's Functional Genomics Core Facility according to the protocol suggested by Affymetrix (Santa Clara, CA). The 6 individual samples were submitted in randomly assigned pairs representing tissues from control (WT) and test (TG) animals: WT1, TG1, WT2, TG2, WT3, TG3. Seven μg of total RNA was used to synthesize cDNA. A custom cDNA kit from Invitrogen was used with a T7-(dT)_24 _primer for this reaction. Biotinylated cRNA was then generated from the cDNA using the BioArray High Yield RNA Transcript Kit. The cRNA was then fragmented in fragmentation buffer (5X fragmentation buffer: 200 mM Tris-acetate, pH8.1, 500 mM KOAc, 150 mM MgOAc) at 94°C for 35 minutes before the chip hybridization. Fifteen μg of fragmented cRNA was then added to a hybridization cocktail (0.05 μg/μl fragmented cRNA, 50 pM control oligonucleotide B2, *BioB*, *BioC*, *BioD*, and *cre *hybridization controls, 0.1 mg/ml herring sperm DNA, 0.5 mg/ml acetylated BSA, 100 mM MES, 1 M [Na^+^], 20 mM EDTA, 0.01% Tween 20). Ten μg of cRNA was used for hybridization. Each sample was hybridized to a separate oligonucleotide array (Affymetrix MG-U74Av2) for 16 hours at 45°C in the GeneChip Hybridization Oven 640. The arrays were washed and stained with R-phycoerythrin streptavidin in the GeneChip Fluidics Station 400. After this, the arrays were scanned with a Hewlett Packard GeneArray Scanner. Affymetrix GeneChip Microarray Suite (MAS) 5.0 software was used for scanning and basic analysis. Sample quality was assessed by examination of 3' to 5' intensity ratios of certain genes.

The MG-U74Av2 array contains probes for 12,488 genes and each gene is represented by multiple (~16) probe pairs. Each probe pair consists of a perfect match (PM) and a mismatch (MM) 25-base oligonucleotide. The MM oligonucleotide is identical in sequence to the PM except for a single mismatch at the center (13^th^) base position. This probe pairing design helps identify and subtract non-specific hybridization and background signal. Probes represent unique regions of the gene, allowing for specific detection of individual gene transcripts, even among genes from the same family. Each array also contains probe pairs for controls added to the sample prior to hybridization as indicated above.

For each probe pair, the MM (non-specific) signal is subtracted from the PM (total) signal. The average of these differences represents the final "signal" and is reflective of the level of expression of the gene. The next step is "global scaling", a form of normalization in which the intensities of the probe sets are scaled to an average representing all probe sets, making different arrays of the same experiment comparable. Finally the MAS 5.0 program creates the user's file (so-called .CHP file), which can be of two forms: single array file or comparison file.

For *single array analysis*, MAS 5.0 calculates statistical significance for the average difference between probe pairs and generates the detection calls Absent, Marginal, or Present for each gene transcript. A value of p > 0.06 indicates that the gene is not detected at a level significantly different from background and is thus designated as Absent; a p value between 0.06 and 0.04 results in a Marginal call; and a p < 0.04 indicates Present. The *comparison analysis *in MAS 5.0 [14] estimates the magnitude and direction of changes of a transcript when two conditions are compared directly (experiment versus control). In order to do this, the software first calculates a probe log ratio (PLR) for each probe in the probe set by subtracting the logarithm of the signal in the control array from that in the experimental array, resulting in 16 PLR values per gene. The Signal Log Ratio (SLR) is the weighted mean of these 16 values and is associated with a p value and a change call of Decreased, Unchanged, or Increased; the fold change can be calculated as the exponential of the log ratio. This strategy cancels out differences (variability) due to different probe binding coefficients and removes much of the proportional relationship between random error and signal intensity [15]; it is therefore a more accurate estimate of differential expression than comparisons made from single-array analyses where the probe pairs are first averaged so that only a single pair of values is used for comparison.

The text files containing signals, p-values, and detection calls were imported to GeneSpring 6.2 (Silicon Genetics, Redwood City, CA) software. In GeneSpring, single-array expression files were normalized to the median of the same array. All data were filtered so that only signals designated Present or Marginal (p = 0.06) were included in further analysis. Lists of genes up- or down-regulated by at least a factor of 2 in TG mice as compared to WT were constructed in GeneSpring using the comparison files. These two lists were further analyzed using the on-line software OntoExpress [16,17]. OntoExpress constructs functional profiles using gene ontology terms for the following categories: biochemical function, biological process, cellular role, cellular component, molecular function, and chromosome location. Finally, the lists of up- and down-regulated genes were imported into PathwayAssist (PA; Stratagene, LaJolla, CA). PA is a bioinformatics software that combines information from microarray data, scientific literature, and the most important databases to build pathways and connectivity maps showing how genes, proteins, and small molecules interact to mediate cellular processes. "Connectivity" (or "controls") is defined in PA as one of the following 10, user-selectable, functional relationships: Binding, Regulation, Molecular synthesis, Molecular transport, Expression, Protein modification, Cell object control, Chemical reaction, Enzymatic activity, and Cell process control. At the time of this analysis, the database contained over 200,000 objects (proteins and small molecules) and over 100,000 controls (documented interactions).

### Real-time quantitative PCR analysis of representative genes

Quantitative real-time RT-PCR was carried out on 8 different transcripts in a LightCycler (Roche Applied Science, Indianapolis, IN) available in the Laboratories for Reproductive Biology at UNC-CH. One μg of total RNA, extracted from mouse testis and purified as described above, was reverse transcribed to cDNA with oligo (dT)12–18 primer to serve as a template. Forward and reverse primers for the genes of interest were designed and real-time PCR was run according to the manufacturer's instructions. Amplification consisted of 40 cycles at 95°C for 30 sec (denaturation), 55–58°C for 5 sec (annealing), and 72°C for 10 sec (elongation). SYBR Green I fluorescence was used to detect the amplified products. For each quantification, a standard curve was created using suitably diluted appropriate cDNA. Following the PCR amplification, melting curve analysis was performed on the amplified product to ensure the accuracy of quantification. In some cases, this was further confirmed by nucleotide sequencing.

## Results

As shown earlier [9,10], differences in the weight, histological structure, and cellular composition of the testes of WT and TG mice involve only a small percentage (<3%) of the total cell population and these differences in themselves are not expected to introduce a major bias in global gene expression profiles.

The complete and unedited data files generated from the 6 microarrays are available as supplemental material on the web site . The sigle array files are designated as WT1, WT2, WT3, TG1, TG2, and TG3; the comparison files are designated as TG1 vs WT1, TG2 vs WT2, and TG3 vs WT3.

**Figure 1:**
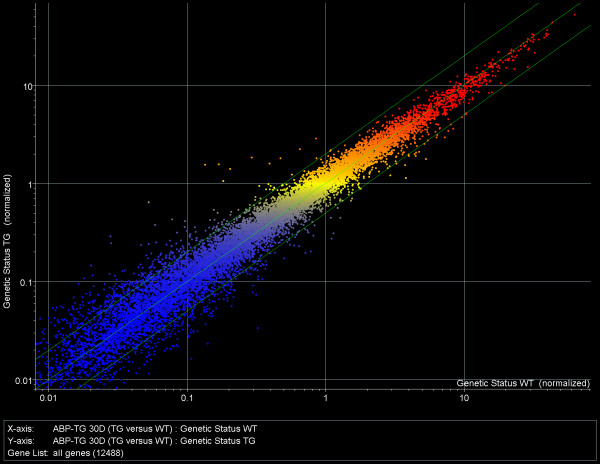
Two-dimensional scatter-plot of the microarray results. Each dot represents average expression values for the same gene from control (wild type, WT) animals (horizontal axis) and from ABP-transgenic (TG) animals (vertical axis) on a log_10 _scale. Genes with similar expression levels appear along the first diagonal (the line y=x); genes with expression levels that are different in the two groups appear above and below this line, respectively; the larger the difference, the farther away the point will be from the y = x line. The two parallel green lines mark the limits for two-fold differences. This identifies *478 genes that are up-regulated *at least 2-fold and *311 genes that are down-regulated *at least 2-fold in the TG animals, compared to the WT.

A scatter-plot summary of the microarray expression data including all genes represented on the MU74Av2 chip (12,488) is shown in Fig. 1. The consistent location of average expression values along the first diagonal (the line for x = y in Fig. 1) indicates that there was no systematic bias between control and experimental groups. As expected, a higher variance is seen at lower expression levels than at higher ones. Fig. 1 also indicates that, although the expression levels of most genes do not differ between the WT and TG testes, a substantial number of genes are expressed at an elevated or reduced level in the TG as compared to the WT testes. When the arbitrary difference of 2-fold (or greater) change was selected, 478 genes were identified as up-regulated and 311 genes were identified as down-regulated in the TG vs. the WT testes.

When a filter was applied to select only those genes whose expression level is significant (p = 0.05) in at least 3 out of the 6 samples (to allow inclusion of genes that are expressed below detection limit in one but not in the other group), a total of 9,810 genes were identified and of these 471 were up-regulated and 305 were down-regulated in the TG vs. the WT samples.

**Figure 2:**
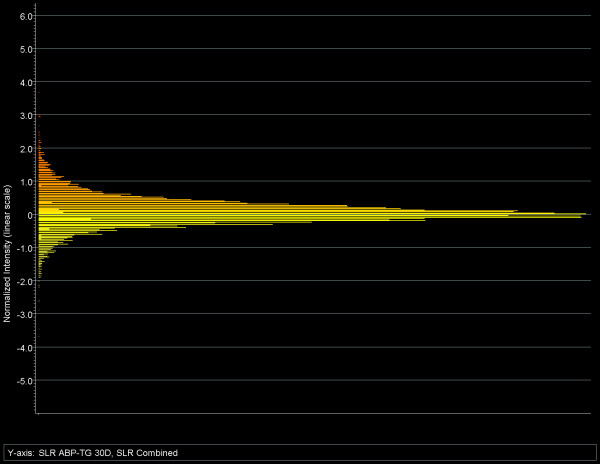
Frequency distribution histogram of the average signal log ratios (SLR) from the three comparison files. The SLR reflects both the direction and the magnitude of changes in gene expression in TG mice compared to WT (for detailed explanation, see the text). The vertical scale is a 2-based log scale used in computing SLR. On this scale, 0 represents no change, +1.0 and -1.0 represent a 2-fold, +2.0 and -2.0 a 4-fold increase and decrease, respectively. This analysis identifies *381 genes as up-regulated *(i.e., those above the horizontal line +1.0) and *198 genes as down-regulated *(below the horizontal line -1.0).

Plotting the combined (SLR) values from the Affymetrix comparison files in a frequency distribution graph allows direct visualization and evaluation of the differential expression of all genes, as shown in Fig. 2. Fig. 2 uses the average of SLRs obtained from the 3 comparison files (TG1 vs. WT1, TG2 vs. WT2, TG3 vs. WT3). On the log2 scale used, 0 represents no change, +1 and -1 represent 2-fold, +2 and -2 a 4-fold increase and decrease, respectively. This histogram also reflects the near-symmetrical distribution of the data around the 0 or "no change" level and allows easy selection of genes with any desired fold change value. Because of the more stringent validation criteria used in the calculation of SLR values (see above), this analysis identified 381 genes (3.05%) that were up-regulated and 198 genes (1.59%) that were down-regulated at least by a factor of 2, respectively. The two gene lists created this way were used for all further analyses presented in this report. The complete lists are available as supplemental material in Additional file 1 and Additional file 2.

The gene lists shown in Additional files 1 and 2, i.e. the genes differentially expressed between the TG and WT groups, were subjected to "functional clustering" according to the Gene Ontology (GO) classification [18], using the on-line software OntoExpress [16,17]. The results are presented in Tables 1 and 2. Only those categories were included in Tables [Table T1]1 and 2 that contained at least 3 transcripts. OntoExpress identified 26 GO categories that were significantly overrepresented in the list of the up-regulated transcripts (Table 1). As seen from Table 1, the largest numbers of transcripts represent those encoding membrane proteins, components of G-protein-coupled receptor signaling pathways, catalytic activities, proteins participating in the immune response, and proteins related to cytoskeleton organization and biogenesis, among many others. Interestingly, among the up-regulated transcripts, a significantly greater number than expected (n = 22, p = 0.00759) were products of genes located on Chromosome 3. From the list of the down-regulated transcripts, OntoExpress identified 15 GO categories that were significantly overrepresented (Table 2). In this group, transcripts of those genes coding for extracellular proteins were enriched most dramatically, followed by those related to proteolysis, cell adhesion, immune response, and growth factor, cytokine, and ion channel activities.

**Table 1 T1:** Gene ontology categories significantly (p < 0.05) overrepresented among the 381 gene transcripts up-regulated in the testis of ABP-transgenic mice*

**P-value**	**Number of transcripts**	**Percent**	**GO category**
0.00708	46	12.07	Membrane
0.00759	22	5.77	Genes located on chromosome 3
0.0449	11	2.89	GPCR signaling pathway
0.03895	11	2.89	Catalytic activity
0.0129	9	2.36	Immune response
0.03625	7	1.84	Peptidase activity
0.03774	7	1.84	Rhodopsin-like receptor activity
0.00798	7	1.84	Cytoskeleton organization and biogenesis
0.00225	6	1.57	Chemotaxis
0.01054	5	1.31	Intermediate filament
0.04126	5	1.31	Serine-type endopeptidase activity
0.00641	5	1.31	Muscle development
0.0152	5	1.31	Lyase activity
1.5E-4	4	1.05	Steroid binding
0.00786	4	1.05	Sodium ion transport
0.02409	4	1.05	Serine-type endopeptidase inhibitor activity
0.00862	3	0.79	Peroxidase activity
<1.0E-5	3	0.79	GP signaling, coupled to IP3 second messenger (phospholipase C activating)
0.04783	3	0.79	Steroid hormone receptor activity
0.03299	3	0.79	Heterotrimeric G-protein complex
0.04461	3	0.79	Ligand-dependent nuclear receptor activity
0.02793	3	0.79	Lipid catabolism
0.03571	3	0.79	Symporter activity
0.00561	3	0.79	Response to oxidative stress
0.03855	3	0.79	Regulation of transcription
4.9E-4	3	0.79	Structural constituent of eye lens

*Categories containing fewer than 3 transcripts were excluded.

Abbreviations: GP: G-protein; GO: Gene ontology; GPCR: G-protein-coupled receptor; IP3: inositol-3-phosphate.

**Table 2:** Gene ontology categories significantly (p < 0.05) overrepresented among the 198 gene transcripts down-regulated in the testis of ABP-transgenic mice*

**P-value**	**Number of transcripts**	**Percent**	**GO category**
0.00838	37	18.69	Extracellular space
0.00203	10	5.05	Extracellular
0.02622	8	4.04	Proteolysis and peptidolysis
0.04705	7	3.54	Cell adhesion
0.00209	7	3.54	Immune response
0.00622	5	2.53	Growth factor activity
0.00566	5	2.53	Cytokine activity
0.04561	5	2.53	Ion channel activity
0.04894	4	2.02	Rhodopsin-like receptor activity
0.00567	4	2.02	Chymotrypsin activity
0.00567	4	2.02	Hormone activity
0.00752	4	2.02	Trypsin activity
0.00000	3	1.52	Positive regulation of MHC class II biosynthesis
0.0105	3	1.52	Monooxygenase activity
0.00219	3	1.52	Blood coagulation

*Categories containing fewer than 3 transcripts were excluded.

**Table 3:** Genes with the highest connectivity scores among all (381) genes up-regulated in the testes of ABP-TG mice.

**Name**	**GenBank ID**	**Connectivity score**	**Fold increase**	**Description**
IL-6	X54542	969	2.52	Cytokine; extracellular
AGT	AF045887	927	2.64	Protease inhibitor; extracellular
MYC	L00039	594	2.25	Oncogene, transcription factor; cell cyle, apoptosis
CAT	M29394	405	2.19	Peroxidase; intracellular
EGR1	AV369921	323	2.46	Transcription factor; differentiation, mitogenesis
NTF3	X53257	144	2.41	Growth factor; extracellular
A2M	AI850558	132	2.14	Protease in hibitor; extracellular
LYZS	M21050	132	2.14	Lysozyme; extrecellular
CFTR	AV374675	123	3.40	Ion channel (cystic fibrosis transmembrane conductance regulator); plasma membrane
SHBG	U85644	122	2.41	Androgen-binding protein/ sex hormone binding globulin; extracellular
PGR	M68915	123	2.19	Progesterone receptor; transcription factor
HAND2	U43715	109	2.19	Heart and neural crest derivatives expressed transcript 2; transcription factor
VDR	AW061016	102	2.41	Vitamin D receptor; transcription factor
GDNF	AV296394	99	2.52	Growth factor; extracellular
RAD51	AV311591	97	7.64	DNA repair; meiosis
BTK	AV227438	91	2.14	Tyrosine kinase; intracellular
MAP2K3	X93150	86	2.41	Signal transduction; intracellular
MME	M81591	85	2.00	Metallo-endopeptidase; plasma membrane
CD36	L23108	80	2.05	Collagen type I receptor; cell adhesion, apoptosis; plasma membrane
HSPA8	AV257761	78	2.19	Heat shock protein; chaperone; intracellular

**Table 4:** Genes with the highest connectivity scores among all (198) genes down-regulated in the testes of ABP-TG mice.

**Name**	**GenBank ID**	**Connectivity Score**	**Fold decrease**	**Description**
MAPK8	AB005663	1000	2.70	Signal transduction; protein kinase
IL-2	K02292	673	3.73	Cytokine; growth factor; extracellular
IL-4	AA967539	681	2.05	Cytokine; humoral defense; extracellular
IL-10	M37897	427	3.32	Cytokine; immune response; extracellular
HGF	X72307	392	2.76	Hepatocyte growth factor; trypsin activity; extracellular
CREB1	X67719	344	2.14	Transcription factor, linked to cAMP signaling
GAST	AV062425	267	2.30	Gastrin; extracellular
MMP2	M843224	179	2.25	Matrix metalloprotease; type IV collagenase; extracellular
CD44	U57611	166	2.41	Hyaluronan receptor; cell surface glycoprotein
F10	AF087644	133	2.96	Coagulation factor X; serine protease; extracellular
MPO	X15313	129	3.32	Myeloperoxidase; antibacterial defence; extracellular
GHRH	M31654	103	2.00	Growth hormone releasing hormone; extracellular
ITIH4	AF023919	91	2.46	Trypsin inhibitor; extracellular
SLC9A	U51112	79	2.46	Ion channel; Na+/H+ exchanger; transmembrane protein
GFAP	X02801	74	2.14	Intermediate filament/structural protein; intracellular
VWF	AI843063	63	2.30	Von Willebrand factor; cell attachment; extracellular
SMARCA-4	AA097203	45	2.25	Transcription factor; chromatin remodeling; nucleus
HCRT	AF019566	38	2.46	Precursor of hypocretin neuropeptides; extracellular
GATA2	AV377670	37	3.18	Transcription factor; development; nucleus
NTRK3	AF035400	34	2.76	Cell surface receptor, non-catalytic isoform

For the initial interaction analysis, the complete lists of genes up- or down-regulated in the transgenic mice (Additional files 1 and 2) were imported to PA as a "new pathway". The program displays the number of interactions ("connectivity score") known for each gene or protein. The 20 genes with the highest scores from each list were selected and are listed in Tables 3 and 4.

**Figure 3:**
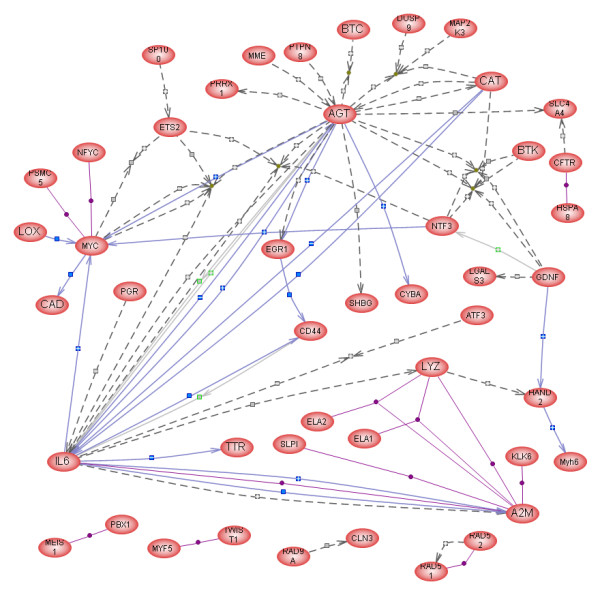
Interactive map (pathway) of 46 genes/proteins created in PathwayAssist^® ^from the list of transcripts up-regulated in the testes of ABP-transgenic mice. AGT, A2M, IL-6, and MYC are "nodes" with multiple connections whereas most other components have only one or two connections. The symbols along the connecting lines indicate the nature of the interaction (Expression: blue square; Regulation: gray square; Molecular transport: gray square with green outline; Protein modification: yellow hexagon; Binding: purple diamond; Promoter binding: green diamond; Molecular synthesis: light blue square with dark blue outline; and Chemical reaction: clear square; stimulation or inhibition is indicated by + or - signs within the symbols).

**Figure 4:**
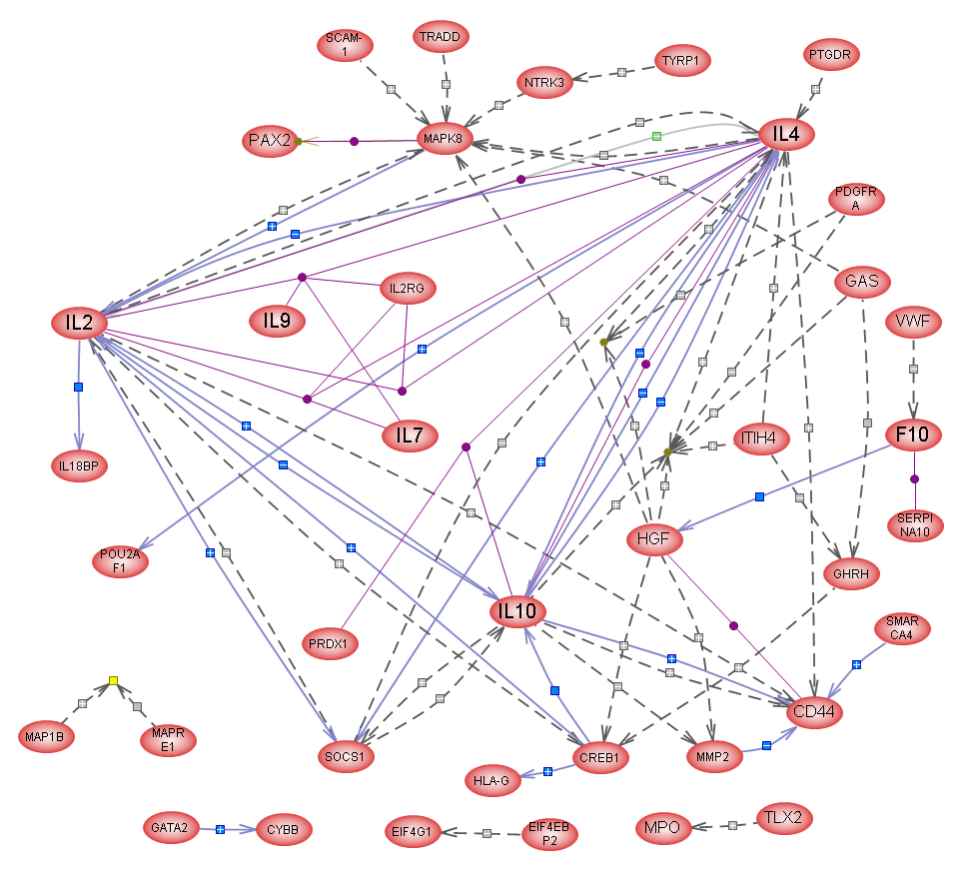
Interactive map (pathway) of 38 genes/proteins created in PathwayAssist^® ^from the list of transcripts down-regulated in the testes of ABP-transgenic mice. Major nodes are IL-2, 4, and 10, MAPK8, SOCS1, CD44, and CREB1. For explanation of symbols, see legend to Fig. 3.

PahwayAssist was also used to create functional (interaction) networks of the up- or down-regulated genes. All the genes belonging to the up-regulated group were displayed, all were selected, and the "Build Pathway" command was used with the option of "Find only direct interactions between selected nodes" to create a new pathway (Fig. 3). This network includes 46 proteins connected by 88 interactions. Four major nodes are evident in this network: Il-6, AGT, MYC, and A2M, which are connected to each other and to other proteins with a large number of interactions; most other proteins have only 1 or 2 interactions within the network. A similar interaction network was created from the list of down-regulated genes (Fig. 4). Of the proteins in this gene list, 38 were directly connected to each other through 72 "contols" or interactions (Fig. 4). This network includes several major nodes (Il-2, -4, and -10, MAPK8, SOCS1, and CREB1). Since up- and down-regulated proteins can interact with each other, we have attempted to combine the two groups and carry out the interaction analysis on the combined list of 579 genes/proteins. The resulting pathway contained 434 nodes and 12,610 interactions and was too large to be reproduced here.

The results of quantitative PCR analysis carried out on selected transcripts representing decreased, unchanged, and increased levels of transcription are presented in Table 5.

**Table 5:** Comparison of expression ratios of selected genes from testes of ABP-transgenic (TG) and wild type (WT) mice as determined by real-time PCR and microarray analysis.

**Name and GenBank Accession ID**	**Primers**	**TG/WT ratio RT-PCR**	**TG/WT ratio microarray**
Androgen receptor M37890	5' GGC CCC CAT CCA AGA CCT ATC 3' 5' GGC TCG GGG AGG CAG CTG 3'	0.95*	1.00
Aromatase P450 D00659	5' GGG CCC CTC ATT TCC CAT GG 3' 5' CCG GTC CAA ATG CTG CTT GAT G 3'	1.13	1.02
17-beta-hydroxysteroid dehydrogenase 1 AF039299	5' GCT CCT CTG GAA TCG GCA TGC 3' 5' CCA CAC GAC CCT CAG TCA CG 3'	1.39	0.91
GAPDS testis-specific isoform U09964	5'GGA TTT GGA CGC ATT GGT CG 3' 5'GAT CTC AAG GTT GTC CAC AAC 3'	-1.82	-2.08
24 kDa major androgen-regulated protein, arMEP24 AV381732	5'GAG TCC CCT TGG CCA TTC TG 3' 5'CCT GTG CGA GTC AAT CTG CTC 3'	3.20	3.65
MYC L00039	5'GAT GTG GTG TCT GTG GAG AAG 3' 5'CCG CAC CCC CCT CCA CAA G 3'	2.10	2.25
SHBG/ABP U85644	5' GCC CTG AGA CAC ATT GAC CCT 3' 5' CAG GGC AGG CAG GAG CG 3'	128.08*	2.41
Testis-specific protein U21673	5' GCC AAA CGC ATC AAT CCC CGG 3' 5' GTC ATC AAA CGG AGG TTC CAG G 3'	0.77*	0.93

* Amplified sequence confirmed by nucleotide sequencing

## Discussion

The results reported in Table 5 confirm the fundamental validity of the microarray analysis with the apparent exception of the SHBG/ABP gene product, for which the expression ratio is found to be 128.08 by RT-PCR and only 2.41 by microarray analysis. The reason for this discrepancy is readily apparent if one considers the greater specificity of the microarray analysis compared to the RT-PCR as used here: the TG mice express an excess of *rat *SHBG/ABP and, although the rat and mouse genes are 89% homologous [19], the multiple probes included in the Affymetrix arrays detect the differences and, as a result, do not hybridize as efficiently with the rat mRNA. The primers used for the RT-PCR, on the other hand, do not distinguish between the two species and reflect, correctly, the *total *(both rat and mouse) ABP mRNA present in the testis. This result is in fact a testimony to the added specificity resulting from the multiple probe set design of the Affymetrix microarrays. The 128-fold increase is consistent with our previously reported measurements based on [3H]-dihydrotestosterone-binding activity [8].

The studies reported here establish that in 30-day old ABP-TG mice there are specific changes in testicular gene expression manifested by a 2-fold or greater increase in the transcripts of 381 genes and a 2-fold or greater decrease in the transcripts of 198 genes, a total of 579 genes or 4.64% of all genes represented on the MU74Av2 array. Based on a search of public databases, the expression of nearly 50% of these 579 genes has not been described in testis to date and only a very few have been described as androgen-regulated. We also report, for the first time, enriched gene ontology categories and interactive networks of gene products that are co-regulated (either induced or repressed) in response to increased levels of intratesticular ABP.

Since we have demonstrated that excess production of ABP in our transgenic animals is associated with normal or reduced levels of androgens [11], and since ABP-bound androgens are regarded biologically inactive [20], it is reasonable to conclude that the changes in gene expression described here reflect a testicular response to a high ABP/low androgen environment. Furthermore, since there is no evidence to date that ABP, either at physiological or at higher than physiological concentrations might have a *direct deleterious *effect on the proliferation and/or differentiation of germ cells in the testis, the impaired spermatogenesis seen in these animals [8,9] is most likely the result of reduced availability of androgens and not the direct effects of excess ABP. Consequently, the ABP-transgenic mouse is a suitable model for studying the effects of chronically reduced levels of androgens on gene expression in testis. Thirty-day old mice were selected for the present analysis because our previous studies had shown that differences in the cellular composition of the testes between TG and WT were minimal (<3%) at this age. Yet, since the first wave of spermatogenesis is already well under way (round spermatids are present in large numbers, and a few elongated spermatids just start to appear [9]), most of the regulatory mechanisms operational in the adult should already be in place.

The complex molecular events that lead to the decline of spermatogenesis following withdrawal of androgens *in vivo *(for a review, see ref. 3) are believed to be initiated in SC, a step that is likely to involve transcriptional mechanisms resulting from reduced activation of AR; the signals generated this way in SC are then greatly amplified (see below) and subsequently delivered to germ cells either through SC surface specializations (membrane proteins, receptors, junctional complexes, ion channels) or via secreted signaling molecules. One of the difficulties in understanding these events stems from the fact that only two genes are known to date that are expressed in SC and respond *directly *to androgens (i.e., possess androgen response elements), the homeobox gene PEM [21] and the protooncogene MYC [22]. Perhaps for this reason, and because of the very complex cellular interactions required for normal spermatogenesis [23,24], previous attempts to identify androgen-regulated gene expression in testis with traditional methods were largely unsuccessful [3].

The first logical step in analyzing differential gene expression is to find the genes with significantly increased or decreased expression in the experimental group compared to controls [25]. As customary in similar studies, a two-fold or greater change (decrease or increase) was arbitrarily defined as the "minimum significant change" for the purposes of the present analysis. (This is not meant to imply that smaller fold changes may not be biologically significant.) The use of the combined SLR values instead of single-array expression data helped to reduce false positives frequently encountered at low levels of expression in single-array analysis [15,16].

As described above and reported in Additional Files 1 and 2, the fold change filter identified 381 increased and 198 decreased gene transcripts in the TG samples as compared to the WT. This simple analysis has thus identified two groups of genes that are *co-expressed *and are, therefore, in some way *co-regulated *in these samples. It is interesting to note that, after a *reduction *of androgen levels, the expression of a larger number of genes is *increased *and that of only a smaller number is decreased. This tendency for up-regulation after androgen withdrawal is consistent with the findings of Griswold's group [26] in genetically hypogonadal mice, in which T treatment generally repressed, rather than stimulated, gene expression. This phenomenon may represent a compensatory mechanism activated in T-deprived Sertoli cells or some of the activated genes may actually be negative regulators of Sertoli cell functions required for the support of spermatogenesis.

Functional analysis of groups of genes as large as 381 and 198, respectively, is still difficult by the traditional "gene by gene" approach. Traditional clustering algorithms available in GeneSpring (dendrograms, K-means, self-organizing maps, etc.) did not seem to reveal biologically meaningful clusters of genes in the present study.

The GO annotations [18] together with statistical evaluation of the level of representation of each GO category by OntoExpress [16,17] provide a standardized, clear, and effective way of classifying gene expression data into biologically meaningful classes. When the differentially up- or down-regulated genes (Additional Files 1 and 2) were subjected to this analysis, several functionally defined groups were identified (Tables 1 and 2). Prominent among these are membrane proteins, various groups of intracellular signaling molecules, proteolytic enzymes, and proteins related to the immune response and cytoskeletal organization (up-regulated), and extracellular structural and messenger molecules and those related to cell adhesion (down-regulated). Detachment of germ cell clusters from SC is a known consequence of both experimental withdrawal of testosterone [27] and hypersecretion of ABP [8].

Both differentially expressed (up- or down-regulated) groups include several *ion channels*. Among the up-regulated ion channels are several potassium channels (Kcne1, Irk1, Kcc1), sodium channels (BLINaC, Slc4a4, Ntcp), the cAMP-regulated chloride channel CFTR, and the water transporter Aqp4. Seminiferous tubule fluid in normal rats contains ten times higher potassium but lower sodium concentrations than blood; the secretion of the fluid and the maintenance of the ion gradients depend on SC [28] and are androgen-regulated [29,30]. Among the down-regulated genes are those coding for calcium entry channel TRP6, a voltage-gated potassium channel β-subunit KVB1, and the G-protein-coupled inward rectifying potassium channel 1 (GIRK1). Mutations of the second subunit of this channel, GIRK2, are responsible for the *weaver *phenotype characterized by degeneration of the granule cells of the cerebellum, testicular germ cells, and Sertoli cells [31].

The results of the "ontological clustering" with the help of Onto-Express [16,17] (Tables 1 and 2) bring into focus the biologically important changes within the large number of unorganized data generated by the microarray analysis. These results represent a specific combination or pattern of cellular functions that change in response to a particular stimulus (in this case, reduction of bioavailable androgens), ultimately resulting in a particular phenotype. Thus, these results are the *gene expression profile of that phenotype*.

Most cellular functions are carried out by proteins interacting with each other and with a variety of small molecules. The software PathwayAssist is capable of identifying such interactions based on evidence available in public databases. In an attempt to further narrow our focus from functional groups of genes to individual genes and their transcripts, we reasoned that proteins with greater number of interactions must have a greater impact on cellular functions than proteins with lower number of interactions. A similar strategy was used by Yonan et al. [32] to identify genes or gene variants that may contribute to autism.

The strategy based on connectivity scores identified a number of genes as major candidates that may mediate androgens' actions on spermatogenesis. Among the up-regulated high-impact genes (Table 3), 7 code for secreted enzymes or signaling molecules (IL-6, AGT, NTF3, A2M, LYZS, SHBG, and GDNF), 6 for transcription factors and a DNA repair molecule (MYC, EGR1, PGR, VDR, HAND2, RAD51), 3 for cell surface proteins (CFTR, MME, CD36), 2 for intracellular signal transducers (MAP2K3, BTK), and 2 for intracellular proteins related to stress response (CAT, HSPA8). Only the most important of these genes will be briefly discussed below.

Among the secreted signaling molecules, ***IL-6 ***is produced in the testis by both SC and Leydig cells in response to a number of stimuli [33]. Its receptor, gp130, is also expressed in SC [34] and activation of the receptor results in STAT-1 and STAT-3 phosphorylation and subsequent activation of several transcription factors [35]. IL-6 has a number of effects on seminiferous epithelial function, including stimulation of transferrin production by SC [36], and inhibition of meiotic DNA synthesis in pre-leptotene spermatocytes [37]. It has been suggested that IL-6 expression is a downstream target for JNK (MAPK8) in myoblasts [38]. ***A2M ***(alpha-2-macroglobulin) is a serum protein secreted by SC. It is a major nonspecific protease inhibitor of the seminiferous epithelium, secreted in a stage-specific manner, and controlled in part by FSH [23,39]. It also functions as a cytokine binding/transporting molecule [40] and is thus capable of modulating the actions of proteases and growth factors in the seminiferous epithelium. ***LYZS ***encodes lysozyme, whose natural substrate is the bacterial cell wall peptidoglycan, cleaves the beta [1-4] glycosidic linkages between N-acetylmuramic acid and N-acetylglucosamine. It has been proposed that lysozyme may function as a receptor/binding protein for N-acetylglucosamine [41]. Through this interaction, lysozyme may regulate germ cell-SC attachment and germ cell survival [42].

***MYC***, a member of the helix-loop-helix/leucine zipper superfamily, is expressed during proliferation in a wide variety of adult tissues including the testis. In transgenic rats expressing c-myc, spermatogenesis is arrested at the level of primary spermatocytes and apoptotic DNA-fragmentation increases [43]. C-myc is an androgen-regulated gene expressed in SC [22] and highly expressed c-myc can *repress *the activity of a large number of other genes [44]. Increased testicular c-myc mRNA expression was reported in adult rats treated with the anti-androgen flutamide [45]. Thus the increased expression of c-myc in the ABP-TG mice may contribute significantly to the defect in spermatogenesis seen in these mice. (It is interesting that the only other SC gene that responds to androgens directly, PEM, was not differentially expressed in our samples.)

The ***CD36 ***gene product, a membrane-associated glycoprotein, functions as a receptor for thrombospondin and type-I collagen and a facilitator for membrane fatty acid transport. It plays major roles as an adhesion molecule and as a scavenger receptor implicated in cellular lipid metabolism and in the phagocytosis of residual bodies and apoptotic germ cells by SC [46].

Among the 20 down-regulated genes listed in Table 4, as many as 12 code for secreted signaling molecules. These include three interleukins (2, 4, and 10), hepatocyte growth factor, gastrin, a matrix metalloprotease MMP2, myeloperoxidase, two coagulation factors, VWF and F10, a trypsin inhibitor (ITIH4), GHRH, and hypocretin, the precursor protein for the neuropeptides orexin 1 and 2. One gene (MAPK8) is involved in intracellular signal transduction, 3 code for transcription factors (CREB1, SMARCA-4, and GATA2), 3 for cell surface molecules (CD44, SLC9A, and NTRK3), and one for the intermediate filament-structural protein GFAP.

Il-2, -4, and -10 are generally proliferation promoting cytokines, but their site of production and role in testis is not clear. **Il-2 **has been described as a potent inhibitor of Leydig cell steroidogenesis [47]. ***HGF ***(hepatocyte growth factor) is a pleiotropic cytokine apparently produced by peritubular cells of the testis [48]. Its receptor, c-met, is expressed in SC. The HGF-c-met system is required for the formation of seminiferous tubules during development [49]. HGF can promote cell adhesion by up-regulating CD44 in tumor cells [50,51] and our data suggest that a similar relationship may exist in the testis. The ***MMP2 ***gene encodes an enzyme which degrades type IV collagen, the major structural component of basement membranes. Both active MMP2 and its precursor have been detected in SC cultures and their production appears to depend on FSH [52]. MMP2, together with other proteases and protease inhibitors, plays an important role in the maintenance and restructuring of SC tight junctions and the blood-testis barrier, structures whose integrity is indispensable for normal spermatogenesis. ***MAPK8 (JNK)***, an intracellular signal transducer, has the highest connectivity score among all down-regulated genes. MAPK8 is activated by various extracellular stimuli and targets specific transcription factors, thereby mediating immediate-early gene expression in response to cell stimuli. It is a component of the integrin-linked signaling cascade [53]. (C-jun expression was also reduced in our TG animals albeit only by a factor of 0.4.)

Among the three down-regulated transcription factors listed in Table 4, ***CREB1 ***is expressed in SC where it is known to activate a number of genes required for normal spermatogenesis [e.g., [54,55]]. Since CREB1 is down-regulated in the ABP-TG mice, its role in mediating the effects of androgen withdrawal on spermatogenesis in these mice may be significant. The involvement of MAPK and CREB in *non-traditional*, rapid androgen signaling in SC has recently been demonstrated [56,57]. Although the ABP-TG mouse as an experimental model does not allow us to discern rapid effects of androgen withdrawal, it is possible that the same, cell-surface mediated mechanisms remain operational even in a chronic androgen-depletion model. This possibility is reinforced by the large number of signal transduction-related genes whose expression is up- or down-regulated in the ABP-TG mice (see Tables 1, 2, 3, 4, and Figs. 3 and 4).

The interaction networks presented in Figs. 3 and 4 provide additional insights into the changes in testicular gene expression resulting from reduced androgen levels in the testis. Among the major nodes, the transcription factors CREB1 (down-regulated) and MYC (up-regulated) stand out representing perhaps the most important initial phases of the response to reduced androgen levels; next, it is apparent that paracrine factors, such as the interleukins 2, 4, 6, and 10, angiotensinogen, alpha-2-macroglobulin, and SOCS1, a negative regulator of cytokine signaling, play important roles in the response. MAPK8 stands out among the intracellular signaling molecules as also playing a central role. Figs. 3 and 4 identify 46 and 38 genes (gene products), respectively, that are not only co-regulated in the ABP-TG mice but are also capable of functionally interacting with each other. This conclusion is reinforced and extended by the results from the combined analysis of all 579 differentially expressed genes, resulting in identification of 434 nodes and 12,610 interactions. The extensive connectivity between these genes indicates that they indeed form a coherent network that reflects and determines the differences between the TG and the WT animals.

Our results suggest a number of important avenues for future research. These include detailed characterization of the identified single genes or relatively small groups of functionally related genes in terms of their involvement in mediating androgens' actions on spermatogenesis. First, it will be important to establish which of the implicated genes are actually expressed in SC. For some of the genes, this is already known (see above); for others, specific localization studies targeting their mRNA or protein products may be performed. In-depth analysis of transcription factors whose expression is significantly affected by androgen withdrawal appears to be an important first step in untangling the complex changes we have observed. Data mining of the extensive interactions among the differentially expressed genes is also likely to provide new functional insights. Whereas our present animal model represents chronic, ongoing androgen deficiency, it will be important to conduct acute and time-resolved experiments by artificial depletion of endogenous androgens followed by restoration of physiological androgen levels within the testis. Such in vivo manipulations of androgen status may be followed by using isolated SC as a source of RNA for microarray studies.

## Conclusion

We report a complex pattern of altered gene expression in mouse testis containing high levels of ABP and low levels of bioavailable androgens. Gene ontology and connectivity analyses of the differentially regulated genes have identified relevant biological processes as well as key individual genes. They include genes whose products function as transcription factors, extra- and intracellular signaling molecules, most prominently cytokines, and extracellular enzymes with the potential of regulating cell-to-cell attachment and cytokine transport. One of only two genes known to be expressed by SC *and *directly respond to androgens, MYC, has been identified as a critical component of the up-regulated network (Fig. 3, Table 2), whereas CREB1, known to be essential for spermatogenesis, was down-regulated. Several ion channels, potential regulators of the extracellular environment within the seminiferous tubules, have also emerged as possible targets of androgen action. Identification of androgens' molecular targets in the testis will lead to better understanding of the physiological control of spermatogenesis, and potentially to the development of new therapeutic and contraceptive approaches.

## Supplementary Material

Additional File 1 This is a Table listing all of the 381 genes whose expression was up-regulated by a factor of 2 or more in the testes ABP-transgenic mice as compared to controls. Click here for file

Additional File 2 This file is a Table listing all of the 198 genes whose expression was down-regulated by a factor of 2 or more in the testes of ABP-transgenic mice as compared to controls. Click here for file
