# Supplementary material for: Microarray analysis of androgen-regulated gene expression in testis: the use of the androgen-binding protein (ABP)-transgenic mouse as a model
Source: Reprod Biol Endocrinol. 2005 Dec 9;3:70. doi: 10.1186/1477-7827-3-70 (PMC1327675; doi:10.1186/1477-7827-3-70)
Supplement: Additional File 1 — This is a Table listing all of the 381 genes whose expression was up-regulated by a factor of 2 or more in the testes ABP-transgenic mice as compared to controls. [file 1477-7827-3-70-S1.doc]

| **AffyID** | **Fold Increase** | **Common Name** | **GenBank ID** | **Description** |
| --- | --- | --- | --- | --- |
| 93122_at | 84.45 | Aeg-1; Crisp1; Aeg1; CRISP-1 | M92849 | Mouse acidic epididymal glycoprotein (Aeg-1) mRNA, complete cds.; cysteine-rich secretory protein 1 |
| 98334_at | 12.70 | CRTAM; Crtam | AF001104 | type I transmembrane protein; immunoglobulin supergene family member; Mus musculus class I MHC-restricted T cell associated molecule (CRTAM) mRNA, complete cds.; cytotoxic and regulatory T cell molecule |
| 160728_r_at | 8.00 | D8Ertd69e | AW048694 | UI-M-BH1-amy-a-02-0-UI.s1 NIH_BMAP_M_S2 Mus musculus cDNA clone UI-M-BH1-amy-a-02-0-UI 3', mRNA sequence.; DNA segment, Chr 8, ERATO Doi 69, expressed |
| 101288_at | 7.82 | Fv4; Fv-4; Akvr-1 | M33884 | env gp70; Mouse endogenous retrovirus in Fv4 locus, complete cds, clone pFv4. |
| 102378_at | 7.64 | KRAG; Sspn; Krag | U02487 | coamplified with Ki-ras in Y1 adrenal carcinoma; Mus musculus LAF putative membrane protein (KRAG) mRNA, complete cds.; sarcospan |
| 162232_r_at | 7.64 | Rad51 | AV311591 | AV311591 RIKEN full-length enriched, 8 days embryo Mus musculus cDNA clone 5730599A20 3' similar to D13473 Mouse mRNA for Rad51 protein, mRNA sequence. |
| 162196_f_at | 6.28 | Itmap1 | AV059956 | AV059956 Mus musculus pancreas C57BL/6J adult Mus musculus cDNA clone 1810060N24, mRNA sequence. |
| 94224_s_at | 5.66 | Ifi205; D3; D3cDNA | M74123 | Mus musculus (strain C57Bl/6) mRNA sequence.; interferon activated gene 205 |
| 160632_at | 5.46 | Prkcn | AW124627 | UI-M-BH2.1-apw-b-08-0-UI.s1 NIH_BMAP_M_S3.1 Mus musculus cDNA clone UI-M-BH2.1-apw-b-08-0-UI 3', mRNA sequence.; protein kinase C, nu |
| 104000_at | 5.28 | 2210023G05Rik | AI181346 | ub83d07.r1 Soares_thymus_2NbMT Mus musculus cDNA clone IMAGE:1395085 5' similar to gb:X53176 Mouse integrin alpha-4 mRNA (MOUSE);, mRNA sequence.; Mus musculus transcribed sequence with weak similarity to protein ref:NP_083693.1 (M.musculus) RIKEN cDNA 9030605E16 [Mus musculus] |
| 161453_r_at | 5.16 | Grsf1 | AV293460 | AV293460 RIKEN full-length enriched, 6 days neonate head Mus musculus cDNA clone 5430437L11 3', mRNA sequence. |
| 94061_at | 4.92 | Crip1; CRHP; CRP1 | M13018 | cysteine-rich intestinal protein (CRIP); Mouse cysteine-rich intestinal protein (CRIP) mRNA, complete cds.; cysteine-rich protein 1 (intestinal) |
| 95532_at | 4.70 | AU015195 | AA691059 | vr67b11.s1 Knowles Solter mouse 2 cell Mus musculus cDNA clone IMAGE:1125693 5', mRNA sequence.; Mus musculus similar to 2-cell-stage, variable group, member 3; 2-cell-stage, variable group, member 1 (LOC236374), mRNA |
| 162465_i_at | 4.70 | BC012312 | AV062451 | AV062451 Mus musculus small intestine C57BL/6J adult Mus musculus cDNA clone 2010003F18, mRNA sequence. |
| 161305_r_at | 4.59 | Usp4 | AV350431 | AV350431 RIKEN full-length enriched, adult male cerebellum Mus musculus cDNA clone 6530431K09 3' similar to L00681 Mus musculus Unp mRNA, mRNA sequence. |
| 162269_at | 4.49 | Smyd2 | AV363431 | AV363431 RIKEN full-length enriched, 15 days embryo male testis Mus musculus cDNA clone 8030423H18 3', mRNA sequence. |
| 94687_at | 4.49 | Foxb1; C43; Mf3; Twh; Fkh5; Foxb1a; Foxb1b; Hfh-e5.1 | U90538 | TWH; transcription factor; Mus musculus winged-helix protein mRNA, complete cds.; forkhead box B1 |
| 96657_at | 4.49 | SSAT; Sat1; SSAT | L10244 | putative; Mouse spermidine/spermine N1-acetyltransferase (SSAT) mRNA, complete cds.; spermidine/spermine N1-acetyl transferase 1 |
| 94069_r_at | 4.29 | 2610019A05Rik | AW230066 | up25b01.y1 NCI_CGAP_Mam2 Mus musculus cDNA clone IMAGE:2655337 5', mRNA sequence.; RIKEN cDNA 2610019A05 gene |
| 160367_at | 4.19 | 1110003B01Rik | AW125223 | UI-M-BH2.1-aps-d-01-0-UI.s1 NIH_BMAP_M_S3.1 Mus musculus cDNA clone UI-M-BH2.1-aps-d-01-0-UI 3', mRNA sequence.; RIKEN cDNA 1110003B01 gene |
| 162200_r_at | 4.09 | Agr2 | AV062476 | AV062476 Mus musculus small intestine C57BL/6J adult Mus musculus cDNA clone 2010003G24, mRNA sequence. |
| 93458_at | 4.09 | C330003B14Rik | AA501129 | vh72h02.r1 Knowles Solter mouse inner cell mass Mus musculus cDNA clone IMAGE:892563 5', mRNA sequence.; RIKEN cDNA C330003B14 gene |
| 161880_r_at | 4.00 | Trappc5 | AV087390 | AV087390 Mus musculus tongue C57BL/6J adult Mus musculus cDNA clone 2310030H03, mRNA sequence. |
| 160639_at | 3.91 | Hpn | AF030065 | serine protease; Mus musculus serine protease hepsin mRNA, complete cds. |
| 162211_r_at | 3.91 | 9830126M18 | AV379320 | AV379320 RIKEN full-length enriched, adult male epididymis Mus musculus cDNA clone 9230005C21 3', mRNA sequence. |
| 160375_at | 3.91 | ca3; Car3; Car-3 | AJ006474 | Mus musculus ca3 gene 5'UTR and exon 1. |
| 162367_f_at | 3.82 | Sec13l-pending | AV233977 | AV233977 RIKEN full-length enriched, 0 day neonate skin Mus musculus cDNA clone 4632428D03 3', mRNA sequence. |
| 92995_at | 3.73 | Vsnl1; VILIP; Vnsl1 | D21165 | Mouse mRNA for neural visinin-like Ca2+binding protein type 1 (NVP-1), complete cds.; visinin-like 1 |
| 161213_r_at | 3.73 | Phf7 | AV264887 | AV264887 RIKEN full-length enriched, adult male testis (DH10B) Mus musculus cDNA clone 4930503L15 3', mRNA sequence. |
| 93515_at | 3.65 | Cdh16 | AF016271 | Mus musculus Ksp-cadherin (Cdh16) mRNA, complete cds.; cadherin 16 |
| 99195_at | 3.65 | 1110018D06Rik | AW120734 | UI-M-BH2.3-any-b-11-0-UI.s1 NIH_BMAP_M_S3.3 Mus musculus cDNA clone UI-M-BH2.3-any-b-11-0-UI 3', mRNA sequence.; Mus musculus 16 days neonate thymus cDNA, RIKEN full-length enriched library, clone:A130028M21 product:hypothetical Arginine-rich region containing protein, full insert sequence |
| 94122_at | 3.65 | MYOC; Myoc; TIGR; GLC1A | AF041335 | myocilin; Mus musculus trabecular meshwork induced glucocorticoid protein (MYOC) gene, exon 3 and complete cds. |
| 96770_at | 3.65 | Dnase1l2 | AA607761 | vo08f08.r1 Stratagene mouse skin (#937313) Mus musculus cDNA clone IMAGE:1040871 5' similar to SW:DRN1_HUMAN P24855 DEOXYRIBONUCLEASE I PRECURSOR ;, mRNA sequence.; deoxyribonuclease 1-like 2 |
| 161986_f_at | 3.65 | Gpx5 | AV381732 | AV381732 RIKEN full-length enriched, adult male epididymis Mus musculus cDNA clone 9230118F08 3' similar to X53780 Mouse mRNA for 24kDa major androgen regulated protein, arMEP24, mRNA sequence.; glutathione peroxidase 5 |
| 100672_at | 3.65 | Myo5a; d; Dbv; MVa; flr; MyoVA; Sev-1; flail; d-120J; dilute | M33467 | dilute gene; Mouse dilute lethal-20J (d-l20J) deletion breakpoint fusion fragment. |
| 161185_i_at | 3.56 | Klf4 | AV235936 | AV235936 RIKEN full-length enriched, 10 day neonate skin Mus musculus cDNA clone 4732408E04 3' similar to L26292 Rattus norvegicus (clone 59) FSH-regulated protein mRNA, mRNA sequence. |
| 102925_at | 3.56 | Dusp9 | AA285446 | vb82a12.r1 Soares mouse 3NME12 5 Mus musculus cDNA clone IMAGE:763486 5', mRNA sequence.; dual specificity phosphatase 9 |
| 97958_at | 3.48 | 3632413B07Rik | AW122355 | UI-M-BH2.2-aow-a-02-0-UI.s1 NIH_BMAP_M_S3.2 Mus musculus cDNA clone UI-M-BH2.2-aow-a-02-0-UI 3', mRNA sequence.; RIKEN cDNA 3632413B07 gene |
| 162215_f_at | 3.48 | Sdc2 | AV380769 | AV380769 RIKEN full-length enriched, adult male epididymis Mus musculus cDNA clone 9230101P06 3' similar to U00674 Mus musculus NMRI fibroglycan (syndecan-2) gene, mRNA sequence. |
| 101639_r_at | 3.48 | Cyp3a16 | D26137 | Mus musculus mRNA for cytochrome P450IIIA, complete cds.; cytochrome P450, family 3, subfamily a, polypeptide 16 |
| 100061_f_at | 3.48 | Klk6; Kal; Klk1; mGk-6; 0610007D04Rik | M13500 | Mus musculus mGK-6 kallikrein gene, exons 3, 4, 5, and complete cds. |
| 160143_r_at | 3.48 | Sycp3; Sycp3; Cor1; Scp3 | Y08486 | M.musculus Sycp3 gene. |
| 161977_r_at | 3.48 | Scin | AV372912 | AV372912 RIKEN full-length enriched, adult male colon Mus musculus cDNA clone 9030613M01 3' similar to Y13971 Mus musculus mRNA for adseverin(D5), mRNA sequence. |
| 161331_r_at | 3.48 | 0610008F14Rik | AV114328 | AV114328 Mus musculus C57BL/6J 10-day embryo Mus musculus cDNA clone 2610031P09, mRNA sequence. |
| 162162_f_at | 3.40 | 1110033L15Rik | AV356864 | AV356864 RIKEN full-length enriched, adult male adrenal gland Mus musculus cDNA clone 7330442P14 3', mRNA sequence. |
| 161904_f_at | 3.40 | Cftr | AV374675 | AV374675 RIKEN full-length enriched, adult male cecum Mus musculus cDNA clone 9130014K16 3' similar to M69298 Mouse cystic fibrosis transmembrane conductance regulator (CFTR) mRNA, mRNA sequence. |
| 96038_at | 3.32 | Ang | AI840339 | UI-M-AJ0-abd-g-01-0-UI.s2 NIH_BMAP_MOB Mus musculus cDNA clone UI-M-AJ0-abd-g-01-0-UI 3', mRNA sequence.; angiogenin |
| 160899_at | 3.32 | Pcp4; Pcp-4; P16Rimb19 | X17320 | unnamed protein product; put. brain specific antigen (AA 1-62); Mouse pcp-4 gene for putative brain specific antigen.; Purkinje cell protein 4 |
| 98857_at | 3.32 | nex-1; Neurod6; Nex; Atoh2; Math2; Nex1m; Math-2 | U29086 | transcription factor; neuronal helix-loop-helix protein; Mus musculus neuronal helix-loop-helix protein NEX-1 (nex-1) mRNA, complete cds.; neurogenic differentiation 6 |
| 101847_at | 3.25 | Sp100; Sp100; A430075G10Rik | AF040242 | nuclear dot gene; Mus musculus Sp100 gene, exon 8 and partial cds. |
| 93437_f_at | 3.25 | 4632419I22Rik | AI850509 | UI-M-BG1-aih-h-10-0-UI.s1 NIH_BMAP_MSC_N Mus musculus cDNA clone UI-M-BG1-aih-h-10-0-UI 3', mRNA sequence.; RIKEN cDNA 4632419I22 gene |
| 161141_r_at | 3.25 | 6030411F23Rik | AV159605 | AV159605 Mus musculus head C57BL/6J 12-day embryo Mus musculus cDNA clone 3010027O11, mRNA sequence. |
| 102729_f_at | 3.25 | Hsd3b6 | AF031170 | 3beta-HSD VI; Mus musculus 3beta-hydroxysteroid dehydrogenase isoform VI mRNA, complete cds.; hydroxysteroid dehydrogenase-6, delta<5>-3-beta |
| 93954_at | 3.17 | GC-S-beta-1; Gucy1b3 | AF020339 | Mus musculus soluble guanylate cyclase beta-1 subunit (GC-S-beta-1) mRNA, complete cds.; guanylate cyclase 1, soluble, beta 3 |
| 97094_at | 3.17 | Phkg | J03293 | Mouse skeletal muscle phosphorylase kinase, gamma subunit mRNA, complete cds.; phosphorylase kinase gamma |
| 96938_at | 3.17 | Keg1; GS4059; 0610008P16Rik | AB028071 | Mus musculus mRNA expressed in renal proximal tubles.; kidney expressed gene 1 |
| 160977_at | 3.17 | Arhgef5 | AA726063 | vu87c09.r1 Stratagene mouse skin (#937313) Mus musculus cDNA clone IMAGE:1209136 5', mRNA sequence.; Rho guanine nucleotide exchange factor (GEF) 5 |
| 161643_i_at | 3.10 | Cln3 | AV076916 | AV076916 Mus musculus stomach C57BL/6J adult Mus musculus cDNA clone 2210020M22, mRNA sequence. |
| 99377_at | 3.10 | Obp1a; OBP-1a | Y10971 | M.musculus mRNA for odorant binding protein Ia, partial. |
| 160795_at | 3.10 | Scamp1 | AW123662 | UI-M-BH2.1-api-h-08-0-UI.s1 NIH_BMAP_M_S3.1 Mus musculus cDNA clone UI-M-BH2.1-api-h-08-0-UI 3', mRNA sequence.; secretory carrier membrane protein 1 |
| 160560_at | 3.10 | Hbb-b1 | AV107330 | AV107330 Mus musculus liver C57BL/6J 13-day embryo Mus musculus cDNA clone 2510030N06, mRNA sequence.; hemoglobin, beta adult major chain |
| 96999_at | 3.10 | or37a; Olfr155; OR37A; mOR37a; Olfr37a; GA_x5J8B7W5BNN-979337-980296 | AJ133424 | Mus musculus or37a gene. |
| 98807_at | 3.10 | Gnat2; Gnat-2; Tcalpha | L10666 | Mus musculus GTP-binding protein superfamily, G protein alpha-t2 subunit (cone transducin) mRNA, complete cds.; guanine nucleotide binding protein, alpha transducing 2 |
| 97119_at | 3.03 | AI596198 | AI596198 | uk23c08.x1 Sugano mouse embryo mewa Mus musculus cDNA clone IMAGE:1969838 3', mRNA sequence.; Mus musculus transcribed sequences |
| 96221_at | 3.03 | AI429613 | AI606300 | vm55g05.y1 Stratagene mouse Tcell 937311 Mus musculus cDNA clone IMAGE:1002200 5', mRNA sequence.; expressed sequence AI429613 |
| 161100_at | 3.03 | 1110062M06Rik | AV305843 | AV305843 RIKEN full-length enriched, 8 days embryo Mus musculus cDNA clone 5730533F24 3', mRNA sequence.; Mus musculus 18-day embryo whole body cDNA, RIKEN full-length enriched library, clone:1110062M06 product:unknown EST, full insert sequence |
| 102076_at | 3.03 | aj4 | AJ235940 | Mus musculus IgVk aj4 gene. |
| 161466_r_at | 3.03 | Asb3 | AV347947 | AV347947 RIKEN full-length enriched, adult male olfactory bulb Mus musculus cDNA clone 6430629E11 3', mRNA sequence. |
| 99437_at | 3.03 | Ly-6F.1; Ly6f | X70922 | M.musculus Ly-6F.1 gene for neurotoxin homologue, exons 1-2. |
| 93460_at | 3.03 | Acvr1; ALK2; SKR1; Tsk7L; ActR-I; ActRIA; Acvrlk2; D330013D15Rik | L15436 | Mus musculus transforming growth factor-beta type I receptor (Tsk 7L) mRNA, complete cds.; activin A receptor, type 1 |
| 92992_i_at | 2.96 | Sp4 | AI324972 | mm78d11.x1 Stratagene mouse embryonic carcinomaRA (#937318) Mus musculus cDNA clone IMAGE:534549 3', mRNA sequence.; trans-acting transcription factor 4 |
| 95350_at | 2.96 | Ttr; prealbumin | D00073 | Mus musculus gene for prealbumin, complete cds and exon 4. |
| 93168_at | 2.96 | AI852629; AV344025 | AI852629 | UI-M-BH0-aiu-f-07-0-UI.s1 NIH_BMAP_M_S1 Mus musculus cDNA clone UI-M-BH0-aiu-f-07-0-UI 3', mRNA sequence.; expressed sequence AV344025 |
| 98552_at | 2.96 | Pmf1 | AW060657 | UI-M-BH1-anl-b-10-0-UI.s1 NIH_BMAP_M_S2 Mus musculus cDNA clone UI-M-BH1-anl-b-10-0-UI 3', mRNA sequence.; polyamine-modulated factor 1 |
| 104597_at | 2.96 | mGBP-2; Gbp2 | AJ007970 | Mus musculus mRNA for mGBP-2 protein.; guanylate nucleotide binding protein 2 |
| 161699_i_at | 2.96 | Irf6 | AV233700 | AV233700 RIKEN full-length enriched, 0 day neonate skin Mus musculus cDNA clone 4632426J10 3' similar to U73029 Mus musculus interferon regulatory factor 6 (mirf6) mRNA, mRNA sequence. |
| 162266_f_at | 2.96 | F8a | AV359917 | AV359917 RIKEN full-length enriched, adult male eyeball Mus musculus cDNA clone 7530403K21 3' similar to M83118 Mus musculus factor VIII-associated protein (f8a) mRNA, mRNA sequence. |
| 99904_at | 2.96 | Itgb3; CD61 | AF026509 | Mus musculus integrin beta3 subunit mRNA, complete cds.; integrin beta 3 |
| 102704_at | 2.96 | Aqp4; Aqp4; MIWC; mMIWC | U88623 | water channel; Mus musculus aquaporin-4 (Aqp4) mRNA, complete cds.; aquaporin 4 |
| 98565_at | 2.96 | Rps26 | AA755219 | vr92b04.r1 Barstead mouse irradiated colon MPLRB7 Mus musculus cDNA clone IMAGE:1136143 5' similar to gb:X69654 40S RIBOSOMAL PROTEIN S26 (HUMAN);, mRNA sequence.; ribosomal protein S26 |
| 101626_at | 2.96 | Adam7; EAP1; EAPI | AF013107 | member of the disintegrin and metalloprotease family; Mus musculus ADAM7 mRNA, complete cds.; a disintegrin and metalloprotease domain 7 |
| 99236_at | 2.89 | Lcn5; Erabp; MEP10; mE-RABP | U68546 | epididymal secretory protein; retinoic acid binding protein; androgen regulated protein; lipocalin family; Mus musculus mE-RABP minor form protein mRNA, complete cds.; lipocalin 5 |
| 95706_at | 2.89 | Lgals3; L-34; gal3; Mac-2 | X16834 | unnamed protein product; Mac-2 antigen (AA 1-264); Mouse mRNA for Mac-2 antigen.; lectin, galactose binding, soluble 3 |
| 102839_at | 2.89 | TRA1; Plscr1; NOR1; TRA1; Tras1; Tras2; MmTRA1a; MmTRA1b; MuPLSCR2 | D78354 | unknown protein; Mus musculus TRA1 mRNA, complete cds.; phospholipid scramblase 1 |
| 99387_at | 2.89 | Fpr1; FPR | L22181 | Mouse N-formyl peptide chemotactic receptor gene, complete cds. |
| 100575_at | 2.89 | Arhgap4 | AI841645 | UI-M-AO0-acg-d-06-0-UI.s1 NIH_BMAP_MPG Mus musculus cDNA clone UI-M-AO0-acg-d-06-0-UI 3', mRNA sequence.; Rho GTPase activating protein 4 |
| 160579_at | 2.89 | Man1a | AI021125 | ub01f03.r1 Soares_mammary_gland_NbMMG Mus musculus cDNA clone IMAGE:1365725 5', mRNA sequence. |
| 100703_at | 2.89 | Npy2r | D86238 | Mus musculus mRNA for neuropeptideY-Y2 receptor, complete cds.; neuropeptide Y receptor Y2 |
| 101865_at | 2.89 | Pip5k2a | AB009615 | Mus musculus mRNA for type II phosphatidylinositolphosphate kinase-alpha, complete cds.; phosphatidylinositol-4-phosphate 5-kinase, type II, alpha |
| 100704_at | 2.89 | Cklfsf4 | D19397 | MUSGS00767 Mouse 3'-directed Mus musculus domesticus cDNA clone mb1508 3', mRNA sequence.; chemokine-like factor super family 4 |
| 94657_at | 2.89 | D8Wsu26e | AA407378 | EST00688 Mouse 7.5 dpc embryo ectoplacental cone cDNA library Mus musculus cDNA clone C0001E05 3', mRNA sequence.; Mus musculus transcribed sequences |
| 101118_at | 2.83 |  | AA511266 | vh78d08.r1 Knowles Solter mouse E6 5d whole embryo Mus musculus cDNA clone IMAGE:893103 3', mRNA sequence.; Mus musculus transcribed sequences |
| 104606_at | 2.83 | pB7; Cd52; B7; MB7; CLS1; CAMPATH-1 | M55561 | Mouse phosphatidylinositol-linked antigen (pB7) mRNA, complete cds.; CD52 antigen |
| 161810_r_at | 2.83 |  | AV372413 | AV372413 RIKEN full-length enriched, adult male colon Mus musculus cDNA clone 9030605K01 3', mRNA sequence. |
| 103484_at | 2.83 | Pop3-pending | AA619442 | vo84c10.r1 Barstead mouse myotubes MPLRB5 Mus musculus cDNA clone IMAGE:1065810 5', mRNA sequence.; Mus musculus transcribed sequence with weak similarity to protein ref:NP_071713.1 (M.musculus) popeye 2; popeye protein 2 [Mus musculus] |
| 161708_f_at | 2.83 | Mpdz | AV244715 | AV244715 RIKEN full-length enriched, 0 day neonate head Mus musculus cDNA clone 4831433K14 3' similar to AF000168 Mus musculus 9ORF binding protein 1 (9BP-1) mRNA, mRNA sequence. |
| 161727_r_at | 2.83 | Rad52 | AV267115 | AV267115 RIKEN full-length enriched, adult male testis (DH10B) Mus musculus cDNA clone 4930524A15 3' similar to U06837 Mus musculus recombination protein RAD52 homolog mRNA, mRNA sequence. |
| 103004_r_at | 2.83 | Cd44; Cd44; Ly-24; Pgp-1; HERMES; AW121933; AW146109 | U57611 | 3' end of coding region for the major form of soluble CD44; hyaluronan receptor; Mus musculus cell surface glycoprotein CD44 (Cd44) mRNA, partial cds, exon 15 (v10). |
| 162309_at | 2.83 | Lyzs | AV014891 | AV014891 Mus musculus 18-day embryo C57BL/6J Mus musculus cDNA clone 1110056N08, mRNA sequence. |
| 162023_f_at | 2.83 | Thbd | AV364086 | AV364086 RIKEN full-length enriched, 15 days embryo male testis Mus musculus cDNA clone 8030438H11 3' similar to X14432 Mouse mRNA for thrombomodulin, mRNA sequence. |
| 162109_f_at | 2.83 | 2200003J05Rik | AV365046 | AV365046 RIKEN full-length enriched, 16 days embryo lung Mus musculus cDNA clone 8430404N19 3', mRNA sequence. |
| 95043_at | 2.83 | Cyp2c70 | AI047331 | ud65a02.y1 Sugano mouse liver mlia Mus musculus cDNA clone IMAGE:1450730 5' similar to gb:M61853 CYTOCHROME P450 IIC18 (HUMAN); gb:D17674 Mouse mRNA for cytochrome P-450, complete cds (MOUSE);, mRNA sequence.; cytochrome P450, family 2, subfamily c, polypeptide 70 |
| 97997_at | 2.83 | Sfrp1 | U88566 | Mus musculus secreted frizzled related protein sFRP-1 (Sfrp1) mRNA, complete cds.; secreted frizzled-related sequence protein 1 |
| 95310_at | 2.83 | bcn; Btc | L08394 | Mus musculus betacellulin (bcn) mRNA, complete cds.; betacellulin, epidermal growth factor family member |
| 93287_at | 2.83 | Blk; Biklk; Blk | AF048838 | BH3-only pro-apoptotic Bcl-2 family member; death agonist that interacts with Bcl-2 and Bcl-xL; Mus musculus Bik-like killer protein (Blk) mRNA, complete cds.; Bcl2-interacting killer-like |
| 101144_at | 2.76 | Il18r1; Il1rrp | U43673 | homolog of type I IL-1 receptor; putative transmembrane receptor; Mus musculus putative transmembrane receptor IL-1Rrp mRNA, complete cds.; interleukin 18 receptor 1 |
| 95957_at | 2.76 | C77070 | C77070 | C77070 Mouse 3.5-dpc blastocyst cDNA Mus musculus cDNA clone J0025F02 3', mRNA sequence.; Mus musculus transcribed sequence with strong similarity to protein ref:NP_036564.1 (H.sapiens) SET domain, bifurcated 1; KIAA0067 gene product; SET domain, bifurcated, 1 [Homo sapiens] |
| 101410_at | 2.76 | mCPE-R; Cldn4; CEP-R; Cpetr; Cpetr1 | AB000713 | Mus musculus mCPE-R mRNA for CPE-receptor, complete cds.; claudin 4 |
| 161669_r_at | 2.76 | Naca | AV171080 | AV171080 Mus musculus head C57BL/6J 14, 17 day embryo Mus musculus cDNA clone 3200002K23, mRNA sequence. |
| 96643_at | 2.76 | 1600023A02Rik | AW121336 | UI-M-BH2.2-aom-b-07-0-UI.s1 NIH_BMAP_M_S3.2 Mus musculus cDNA clone UI-M-BH2.2-aom-b-07-0-UI 3', mRNA sequence.; RIKEN cDNA 1600023A02 gene |
| 96030_at | 2.76 | Csna | M36780 | alpha-casein precursor; Mouse alpha-casein mRNA, complete cds.; casein alpha |
| 93457_at | 2.76 | gamma-B-crystallin; Crygb; Nop; Cryg-3; DGcry-3 | Z22573 | M.musculus gamma-B-crystallin gene. |
| 101774_at | 2.76 | Col4a4; [a]4(IV) | Z35167 | Mus musculus (Balb/c) mRNA for collagen IV alpha 4 chain.; procollagen, type IV, alpha 4 |
| 98570_at | 2.76 | Naca | AI956211 | ul72g05.y1 Sugano mouse kidney mkia Mus musculus cDNA clone IMAGE:2136152 5' similar to TR:Q60817 Q60817 NASCENT POLYPEPTIDE-ASSOCIATED COMPLEX ALPHA POLYPEPTIDE ;, mRNA sequence.; nascent polypeptide-associated complex alpha polypeptide |
| 97402_at | 2.76 | Temt | M88694 | Mus musculus thioether S-methyltransferase mRNA, complete cds. |
| 97458_at | 2.76 | Kpna6 | AI845935 | UI-M-AK1-aex-f-04-0-UI.s1 NIH_BMAP_MHY_N Mus musculus cDNA clone UI-M-AK1-aex-f-04-0-UI 3', mRNA sequence. |
| 98347_at | 2.76 | cdx 4; Cdx4; Cdx3; Cdx-3; Cdx-4 | L08061 | homeobox motif from 673-853; Mus musculus homeobox protein (cdx 4) mRNA, complete cds.; caudal type homeo box 4 |
| 97736_at | 2.73 |  | AI838823 | UI-M-AL0-abu-f-09-0-UI.s1 NIH_BMAP_MCO Mus musculus cDNA clone UI-M-AL0-abu-f-09-0-UI 3', mRNA sequence.; Mus musculus transcribed sequence with weak similarity to protein ref:NP_081764.1 (M.musculus) RIKEN cDNA 5730493B19 [Mus musculus] |
| 104693_at | 2.70 | Dhtkd1 | AA260145 | va37d08.r1 Soares mouse 3NME12 5 Mus musculus cDNA clone IMAGE:733551 5', mRNA sequence.; dehydrogenase E1 and transketolase domain containing 1 |
| 94999_at | 2.70 | Cubn | AJ010338 | Mus Musculus mRNA for hypothetical protein.; cubilin (intrinsic factor-cobalamin receptor) |
| 161245_r_at | 2.70 | 1810045K06Rik | AV312754 | AV312754 RIKEN full-length enriched, adult male thymus Mus musculus cDNA clone 5830405E16 3', mRNA sequence. |
| 94692_at | 2.70 | Gpx5; Arep | X53780 | Mouse mRNA for 24kDa major androgen regulated protein, arMEP24.; glutathione peroxidase 5 |
| 103560_at | 2.70 | 2210402C18Rik | AW124401 | UI-M-BH2.1-ape-d-04-0-UI.s1 NIH_BMAP_M_S3.1 Mus musculus cDNA clone UI-M-BH2.1-ape-d-04-0-UI 3', mRNA sequence.; RIKEN cDNA 2210402C18 gene |
| 95376_at | 2.70 | Clc1; Clcn1 | AJ011107 | Mus musculus mRNA for 3'UTR of Clc1 gene.; chloride channel 1 |
| 98790_s_at | 2.70 | Meis1; Meis1; Evi8; C530044H18Rik | U33629 | Mus musculus myeloid ecotropic viral integration site-1 (Meis1) mRNA, complete cds.; myeloid ecotropic viral integration site 1 |
| 101223_r_at | 2.70 |  | C77112 | C77112 Mouse 3.5-dpc blastocyst cDNA Mus musculus cDNA clone J0026B10 3', mRNA sequence. |
| 103767_f_at | 2.64 |  | AA675006 | vm71a08.r1 Knowles Solter mouse 2 cell Mus musculus cDNA clone IMAGE:1003670 3', mRNA sequence. |
| 100281_at | 2.64 | Gcl | AW060252 | UI-M-BH1-amv-f-02-0-UI.s1 NIH_BMAP_M_S2 Mus musculus cDNA clone UI-M-BH1-amv-f-02-0-UI 3', mRNA sequence.; germ cell-less homolog (Drosophila) |
| 162277_r_at | 2.64 | Polg2 | AV368016 | AV368016 RIKEN full-length enriched, 16 days embryo lung Mus musculus cDNA clone 8430439M15 3' similar to AF006072 Mus musculus mitochondrial DNA polymerase accessory subunit (MtPolB) mRNA, nuclear gene encoding mitochondrial protein, mRNA sequence. |
| 94969_at | 2.64 | Nfyc | U62297 | Mus musculus transcription factor NF-YC subunit mRNA, complete cds.; nuclear transcription factor-Y gamma |
| 101669_at | 2.64 |  | AA589492 | vl48b07.s1 Stratagene mouse skin (#937313) Mus musculus cDNA clone IMAGE:975445 3', mRNA sequence. |
| 102318_at | 2.64 | Siat8d; PST; PST-1; ST8SiaIV | X86000 | Mus musculus mRNA for N-glycan alpha 2,8-sialyltransferase.; sialyltransferase 8 (alpha-2, 8-sialyltransferase) D |
| 93765_at | 2.64 | Dcpp; p20 | U03711 | Mus musculus Swiss Webster demilune cell-specific protein mRNA, complete cds.; demilune cell and parotid protein |
| 101164_at | 2.64 | ox40l; Tnfsf4; gp34; Ox40l; TXGP1; OX-40L; Txgp1l | U12763 | Mus musculus OX40 ligand (ox40l) mRNA, complete cds.; tumor necrosis factor (ligand) superfamily, member 4 |
| 92240_at | 2.64 | Krtap5-4 | AA739024 | vv66b01.r1 Stratagene mouse skin (#937313) Mus musculus cDNA clone IMAGE:1227337 5', mRNA sequence.; Mus musculus transcribed sequences |
| 101887_at | 2.64 | Agt; Aogen | AF045887 | Mus musculus angiotensinogen precursor, gene, exon 5 and complete cds. |
| 101753_s_at | 2.64 | Lzp-s | X51547 | unnamed protein product; precursor protein (AA -18 to 130); Mouse Lzp-s mRNA for lysozyme P (EC 3.2.1.17).; P lysozyme structural |
| 100406_at | 2.58 | Ptpn5; Step | U28217 | A novel 172 amino acid sequence is present in STEP61 relative to the original STEP46 PTP; Mus musculus protein tyrosine phosphatase STEP61 mRNA, complete cds.; protein tyrosine phosphatase, non-receptor type 5 |
| 161279_f_at | 2.58 | Ha1r-pending | AV291749 | AV291749 RIKEN full-length enriched, 6 days neonate head Mus musculus cDNA clone 5430409I04 3', mRNA sequence. |
| 161838_f_at | 2.58 | 2010015J01Rik | AV042598 | AV042598 Mus musculus adult C57BL/6J testis Mus musculus cDNA clone 1700023F18, mRNA sequence. |
| 94735_s_at | 2.58 | Mcptl; MMCP-L | M57401 | Mus musculus mast cell protease-like protein gene, complete cds. |
| 161660_r_at | 2.58 | AI837181 | AV089517 | AV089517 Mus musculus tongue C57BL/6J adult Mus musculus cDNA clone 2310044H21, mRNA sequence. |
| 94415_at | 2.58 | 6230421P05Rik | AA710439 | vt42e01.r1 Barstead mouse proximal colon MPLRB6 Mus musculus cDNA clone IMAGE:1165752 5', mRNA sequence.; Mus musculus 11 days embryo head cDNA, RIKEN full-length enriched library, clone:6230421P05 product:unknown EST, full insert sequence |
| 95958_at | 2.58 | 5930418K15Rik | AA667702 | vv30h04.r1 Stratagene mouse heart (#937316) Mus musculus cDNA clone IMAGE:1223959 5', mRNA sequence.; RIKEN cDNA 5930418K15 gene |
| 92252_at | 2.58 | Cckar | D85605 | Mouse DNA for cholecystokinin type-A receptor, complete cds. |
| 96101_at | 2.58 | Spint2 | AI847090 | UI-M-AP1-agn-e-05-0-UI.s1 NIH_BMAP_MST_N Mus musculus cDNA clone UI-M-AP1-agn-e-05-0-UI 3', mRNA sequence. |
| 94921_i_at | 2.58 | BC022765 | AA220757 | mv66e10.r1 Soares mouse 3NME12 5 Mus musculus cDNA clone IMAGE:660042 5', mRNA sequence.; cDNA sequence BC022765 |
| 92550_at | 2.58 | keratin 19; Krt1-19; K19; EndoC; Krt-1.19; MGC25344 | M36120 | Mouse keratin 19 gene, complete cds. |
| 102284_at | 2.58 | Crarf; Masp1; Crarf | D16492 | Mouse mRNA for P100 serine protease of Ra-reactive factor (RaRF), complete cds.; mannan-binding lectin serine protease 1 |
| 96588_at | 2.58 | Ptger3; EP3; Pgerep3; Ptgerep3 | D10204 | Mus musculus mRNA for prostaglandin E receptor, EP3 subtype, complete cds.; prostaglandin E receptor 3 (subtype EP3) |
| AFFX-BioB-5_st | 2.55 |  |  | J04423 E coli bioB gene biotin synthetase (-5, -M, -3 represent transcript regions 5 prime, Middle, and 3 prime respectively) |
| 100127_at | 2.52 | Crabp2; Crabp-2 | M35523 | cellular retinoic acid-binding protein; Mouse cellular retinoic acid-binding protein (CRABP-II) mRNA, complete cds.; cellular retinoic acid binding protein II |
| 96017_at | 2.52 | 0610006I08Rik | AA710907 | vt44g01.r1 Barstead mouse proximal colon MPLRB6 Mus musculus cDNA clone IMAGE:1165968 5', mRNA sequence.; RIKEN cDNA 0610006I08 gene |
| 102218_at | 2.52 | Il6; Il-6 | X54542 | Mouse mRNA for interleukin-6.; interleukin 6 |
| 93859_at | 2.52 | Mtif2 | AI875598 | uk50c11.x1 Sugano mouse kidney mkia Mus musculus cDNA clone IMAGE:1972436 3' similar to SW:IF2M_BOVIN P46198 TRANSLATION INITIATION FACTOR IF-2, MITOCHONDRIAL PRECURSOR ;, mRNA sequence.; mitochondrial translational initiation factor 2 |
| 101740_at | 2.52 | Adra1a; [a]1a; Adra1c | AF031431 | transmembrane protein; Mus musculus alpha 1A-adrenergic receptor mRNA, complete cds.; adrenergic receptor, alpha 1a |
| 101815_at | 2.52 | Bmp10 | AF101440 | BMP-10; Mus musculus bone morphogenetic protein 10 (Bmp10) gene, exon 2 and complete cds. |
| 162445_at | 2.52 | Hspa5 | AV351546 | AV351546 RIKEN full-length enriched, 12 days embryo male wolffian duct Mus musculus cDNA clone 6720416G14 3' similar to M30779 Mouse glucose-regulated protein 78 mRNA, mRNA sequence. |
| 102799_at | 2.52 | C4bp | M17122 | C4b-binding protein precursor; Mouse C4b-binding protein mRNA, complete cds.; complement component 4 binding protein |
| 161438_r_at | 2.52 | Aars | AV349170 | AV349170 RIKEN full-length enriched, adult male cerebellum Mus musculus cDNA clone 6530401P05 3', mRNA sequence. |
| 96394_at | 2.52 | D2Ertd357e | C81245 | C81245 Mouse 3.5-dpc blastocyst cDNA Mus musculus cDNA clone J0096D03 3', mRNA sequence.; Mus musculus transcribed sequences |
| 97548_at | 2.52 | Prpf39 | AI155120 | ud59e02.r1 Soares_NMPu Mus musculus cDNA clone IMAGE:1450202 5', mRNA sequence.; PRP39 pre-mRNA processing factor 39 homolog (yeast) |
| 161598_at | 2.52 | Gdnf | AV296394 | AV296394 RIKEN full-length enriched, 8 days embryo Mus musculus cDNA clone 5730441H19 3' similar to U66196 Mus musculus glial derived neurotrophic growth factor (GDNF) mRNA, 3'UTR, mRNA sequence. |
| 92325_at | 2.52 | Ptk7 | AI326889 | mj61d06.x1 Soares mouse embryo NbME13.5 14.5 Mus musculus cDNA clone IMAGE:480587 3', mRNA sequence.; PTK7 protein tyrosine kinase 7 |
| 160865_at | 2.46 | Vldlr | L33417 | Mus musculus very low density lipoprotein receptor (VLDLR), complete cds. |
| 160940_at | 2.46 | Sfrs14 | AI550400 | vx13e02.x1 Soares_thymus_2NbMT Mus musculus cDNA clone IMAGE:1264346 3', mRNA sequence.; splicing factor, arginine/serine-rich 14 |
| 94246_at | 2.46 | Ets2; Ets-2 | J04103 | ets2 protein; Mouse erythroblastosis virus oncogene homolog 2 (ets-2) mRNA, complete cds.; E26 avian leukemia oncogene 2, 3' domain |
| 98823_at | 2.46 | IRK1; Kcnj2; IRK1; Kcnf1; Kir2.1 | AF021136 | Mus musculus inward rectifier potassium channel (IRK1) mRNA, complete cds.; potassium inwardly-rectifying channel, subfamily J, member 2 |
| 162094_f_at | 2.46 | Wtap | AV339425 | AV339425 RIKEN full-length enriched, adult male olfactory bulb Mus musculus cDNA clone 6430507K10 3', mRNA sequence. |
| 101704_at | 2.46 | HNF4G; Hnf4g; NR2A2 | AJ242626 | Mus musculus mRNA for hepatocyte nuclear factor 4 gamma.; hepatocyte nuclear factor 4, gamma |
| 101071_at | 2.46 | Myh6; Myhca; Myhc-a | M76599 | Mouse alpha cardiac myosin heavy chain mRNA, complete cds.; myosin, heavy polypeptide 6, cardiac muscle, alpha |
| 161976_r_at | 2.46 | Hig1-pending | AV372261 | AV372261 RIKEN full-length enriched, adult male colon Mus musculus cDNA clone 9030603D12 3', mRNA sequence. |
| 92299_at | 2.46 | Rbbp2 | AI159504 | vz80a02.r1 Soares_mammary_gland_NbMMG Mus musculus cDNA clone IMAGE:1332746 5' similar to gb:S66431 RETINOBLASTOMA BINDING PROTEIN 2 (HUMAN);, mRNA sequence.; Mus musculus adult male corpora quadrigemina cDNA, RIKEN full-length enriched library, clone:B230332A17 product:retinoblastoma binding protein 2, full insert sequence |
| 162467_r_at | 2.46 | AI256456 | AV063606 | AV063606 Mus musculus small intestine C57BL/6J adult Mus musculus cDNA clone 2010007G06, mRNA sequence. |
| 100381_at | 2.46 | alpha-actin; Acta1; Acts; Acta-2; Actsk-1 | M12347 | Mouse skeletal alpha-actin gene, complete cds. |
| 104658_at | 2.46 | Lifr | D17444 | Mouse mRNA for soluble D-factor/LIF receptor, complete cds.; leukemia inhibitory factor receptor |
| 97678_r_at | 2.46 | Cts7 | AV294670 | AV294670 RIKEN full-length enriched, 6 days embryo Mus musculus cDNA clone 5630400J12 3', mRNA sequence.; cathepsin 7 |
| 161300_r_at | 2.46 | Nudt1 | AV349001 | AV349001 RIKEN full-length enriched, adult male cerebellum Mus musculus cDNA clone 6530401E21 3' similar to D49956 Mouse mRNA for 8-oxo-dGTPase, mRNA sequence. |
| 97541_f_at | 2.46 | H2-D1; H-2D | X00246 | unnamed protein product; put. H-2 histocompatibility antigen fragment (aa 44-358); Mouse mRNA with a Set 1 repetitive element for a class I major histocompatibility complex(MHC) antigen.; histocompatibility 2, D region locus 1 |
| 94906_at | 2.46 | Adh1; Adh-1; ADH-AA; Adh-1e; Adh-1t; Adh-3e; Adh1-e; Adh1-t; Adh3-e | M22679 | alcohol dehydrogenase; Mouse alcohol dehydrogenase class I (ADH-A-2) gene, exon 9. |
| 161802_i_at | 2.46 | Egr1 | AV369921 | AV369921 RIKEN full-length enriched, adult male colon Mus musculus cDNA clone 9030201G05 3' similar to M20157 Mouse Egr-1 mRNA, mRNA sequence. |
| 160770_at | 2.41 | Mvd | AW049778 | UI-M-BH1-anm-d-02-0-UI.s1 NIH_BMAP_M_S2 Mus musculus cDNA clone UI-M-BH1-anm-d-02-0-UI 3', mRNA sequence.; mevalonate (diphospho) decarboxylase |
| 104462_at | 2.41 | Hic1 | AW048074 | UI-M-BH1-alq-a-04-0-UI.s1 NIH_BMAP_M_S2 Mus musculus cDNA clone UI-M-BH1-alq-a-04-0-UI 3', mRNA sequence.; hypermethylated in cancer 1 |
| 161353_r_at | 2.41 | 1110007M04Rik | AV156568 | AV156568 Mus musculus head C57BL/6J 12-day embryo Mus musculus cDNA clone 3000003G13, mRNA sequence.; RIKEN cDNA 1110007M04 gene |
| 99964_at | 2.41 | Vdr | AW061016 | UI-M-BH1-amn-f-06-0-UI.s1 NIH_BMAP_M_S2 Mus musculus cDNA clone UI-M-BH1-amn-f-06-0-UI 3', mRNA sequence.; vitamin D receptor |
| 93313_at | 2.41 | MKK3; Map2k3; MEK3; MKK3; Prkmk3; mMKK3b | X93150 | M.musculus mRNA for MAP kinase kinase 3.; mitogen activated protein kinase kinase 3 |
| 96367_at | 2.41 | C79242 | C79242 | C79242 Mouse 3.5-dpc blastocyst cDNA Mus musculus cDNA clone J0063A12 3', mRNA sequence.; Mus musculus transcribed sequences |
| 96997_at | 2.41 | BLINaC; Accn5; Inac; Blinac | Y19035 | Mus musculus mRNA for amiloride-sensitive Na channel (BLINaC gene).; amiloride-sensitive cation channel 5, intestinal |
| 96993_at | 2.41 | PAX-5 | U56837 | Mus musculus B-cell specific transcription factor (PAX-5) gene, exon 1A, partial cds. |
| 92893_at | 2.41 | Nfia; NF1-A; 1110047K16Rik | D90173 | DNA-binding protein, transcription factor ORF of NFI-B2; Mus musculus mRNA for NFI-B protein, complete cds.; nuclear factor I/A |
| 92420_at | 2.41 | NT-3; Ntf3; NT3; NT-3; Ntf-3 | X53257 | M.musculus NT-3 gene for neurotrophin-3. |
| 100702_at | 2.41 | Shbg; ABP | U85644 | Mus musculus testis-specific androgen-binding protein mRNA, complete cds.; sex hormone binding globulin |
| 97832_at | 2.41 | Cd97 | AA754887 | vu55b10.r1 Soares_mammary_gland_NbMMG Mus musculus cDNA clone IMAGE:1195291 5', mRNA sequence.; CD97 antigen |
| 161523_r_at | 2.41 | Calm2 | AV225659 | AV225659 RIKEN full-length enriched, 18 days pregnant, placenta and extra embryonic tissue Mus musculus cDNA clone 3830431E23 3' similar to M17069 Rat calmodulin (RCM3) mRNA, mRNA sequence. |
| 104232_at | 2.41 | cx31; Gjb3; Cx31; Cnx31; Gjb-3; D4Wsu144e | X63099 | M.musculus mRNA for connexin31.; gap junction membrane channel protein beta 3 |
| 94554_at | 2.41 | Trappc5 | AW120965 | UI-M-BH2.3-aod-d-01-0-UI.s1 NIH_BMAP_M_S3.3 Mus musculus cDNA clone UI-M-BH2.3-aod-d-01-0-UI 3', mRNA sequence.; Mus musculus trafficking protein particle complex 5, mRNA (cDNA clone IMAGE:5362649), with apparent retained intron |
| 160797_r_at | 2.35 | Cbx1 | AW124869 | UI-M-BH2.1-apu-b-02-0-UI.s1 NIH_BMAP_M_S3.1 Mus musculus cDNA clone UI-M-BH2.1-apu-b-02-0-UI 3', mRNA sequence.; chromobox homolog 1 (Drosophila HP1 beta) |
| 98028_at | 2.35 | M-twist; Twist1; Pde; M-Twist | M63649 | Mouse M-twist gene, complete cds. |
| 104748_s_at | 2.35 | Slc1a1; EAAC1; EAAT3; MEAAC1 | D43797 | Mouse mRNA for glutamate transporter MEAAC1, complete cds.; solute carrier family 1, member 1 |
| 101183_at | 2.35 | Syt2 | D37793 | Mouse mRNA for synaptotagminII/IP4BP.; synaptotagmin 2 |
| 96963_s_at | 2.35 |  | L14553 | Ig light chain CC49 precursor; Mus musculus Ig light chain V-region CC49 rearranged gene, exons 1 and 2. |
| 100339_at | 2.35 | Ntcp; Slc10a1; Ntcp | U95131 | alternative transcript; Mus musculus Na/taurocholate cotransporting polypeptide 1 (Ntcp) mRNA, alternatively spliced, complete cds.; solute carrier family 10 (sodium/bile acid cotransporter family), member 1 |
| 92531_at | 2.35 | 3100002M17Rik | AI844545 | UI-M-AJ1-ahf-h-03-0-UI.s2 NIH_BMAP_MOB_N Mus musculus cDNA clone UI-M-AJ1-ahf-h-03-0-UI 3', mRNA sequence.; RIKEN cDNA 3100002M17 gene |
| 92858_at | 2.35 | Slpi | AF002719 | Mus musculus secretory leukoprotease inhibitor gene, complete cds. |
| 92681_at | 2.35 | nS7; Magel2; ns7; nM15; NDNL1; Mage-l2 | AJ243608 | new SMAGE gene, ortholog of the human nM15 gene (AJ243531); Mus musculus mRNA for ns7 protein, ortholog of the human nM15 gene.; melanoma antigen, family L, 2 |
| 102677_at | 2.35 | Arhgdig; Gdi5; RIP2; Rho-GDI2; Rho-GDI-3 | U73198 | Ly-GDI guanine nucleotide dissociation inhibitor; Mus musculus Rho-GDI2 guanine nucleotide dissociation inhibitor mRNA, complete cds.; Rho GDP dissociation inhibitor (GDI) gamma |
| 99530_at | 2.35 | Prlpc1; PLP-Ca | AF090140 | Mus musculus prolactin-like protein-Calpha precursor, mRNA, complete cds.; prolactin-like protein C 1 |
| 94006_at | 2.35 | AZ2; Azi2; AZ2 | AB007141 | Mus musculus AZ2 mRNA, complete cds.; 5-azacytidine induced gene 2 |
| 92857_at | 2.35 | Rpl22 | AI853960 | UI-M-BH0-aiv-d-02-0-UI.s1 NIH_BMAP_M_S1 Mus musculus cDNA clone UI-M-BH0-aiv-d-02-0-UI 3', mRNA sequence. |
| 95973_at | 2.35 | C78878 | C78878 | C78878 Mouse 3.5-dpc blastocyst cDNA Mus musculus cDNA clone J0056G01 3' similar to Mouse IgG receptor (beta-Fc-gamma-RII) gene, exons 1-5, mRNA, mRNA sequence.; Mus musculus transcribed sequences |
| 104707_at | 2.35 | 2010003F10Rik | AA718076 | vu54b11.r1 Soares_mammary_gland_NbMMG Mus musculus cDNA clone IMAGE:1195197 5' similar to TR:Q64302 Q64302 L6 ANTIGEN. ;, mRNA sequence.; RIKEN cDNA 2010003F10 gene |
| 103060_at | 2.35 | Lgi4 | AW060519 | UI-M-BH1-ann-d-11-0-UI.s1 NIH_BMAP_M_S2 Mus musculus cDNA clone UI-M-BH1-ann-d-11-0-UI 3', mRNA sequence.; leucine-rich repeat LGI family, member 4 |
| 99979_at | 2.35 | Cyp1-b1; Cyp1b1; CP1B | X78445 | M.musculus Cyp1-b-1 mRNA for cytochrome P450.; cytochrome P450, family 1, subfamily b, polypeptide 1 |
| 94334_f_at | 2.35 | Ina; NF-66; MGC25352 | L27220 | neuronal intermediate filament protein; Mus musculus neuronal intermediate filament protein (alpha-internexin) gene, complete cds. |
| 161368_r_at | 2.35 | Cyp1b1 | AV237975 | AV237975 RIKEN full-length enriched, 10 day neonate skin Mus musculus cDNA clone 4732424O06 3' similar to U03283 Mus musculus C3H cytochrome P450 (Cyp1b1) mRNA, mRNA sequence. |
| 92740_at | 2.30 | Iga | J00475 | secreted form; Mouse Ig germline D-J-C region alpha gene and secreted tail. |
| 94754_at | 2.30 | Lhx8; L3; Lhx7 | D49658 | Mus musculus mRNA for LIM-homeodomain protein, partial cds.; LIM homeobox protein 8 |
| 98327_at | 2.30 | AU015084 | AA517848 | vh82c12.r1 Knowles Solter mouse E6 5d whole embryo Mus musculus cDNA clone IMAGE:893494 3', mRNA sequence.; Mus musculus transcribed sequences |
| 97746_f_at | 2.30 | Hox-1.4; Hoxa4; Hox-1.4 | X66861 | Mus musculus Hox-1.4 gene. |
| 93522_at | 2.30 | Rad9 | AF045663 | Mus musculus radio-resistance/chemo-resistance/cell cycle checkpoint control protein (Rad9) mRNA, complete cds.; RAD9 homolog (S. pombe) |
| 94511_at | 2.30 | Ssr1 | AI850546 | UI-M-BG1-aij-d-07-0-UI.s1 NIH_BMAP_MSC_N Mus musculus cDNA clone UI-M-BG1-aij-d-07-0-UI 3', mRNA sequence. |
| 98827_i_at | 2.30 | Kif5c; KINN; NKHC; NKHC2; NKHC-2; Kif5a | X61435 | M.musculus mRNA for kinesin heavy chain.; kinesin family member 5A |
| 92356_at | 2.30 | 70zpep; Ptpn8; PEP; 70zpep | M90388 | Mouse protein tyrosine phosphatase (70zpep) mRNA, complete cds.; protein tyrosine phosphatase, non-receptor type 8 |
| 161709_at | 2.30 | Fancc | AV245037 | AV245037 RIKEN full-length enriched, 0 day neonate head Mus musculus cDNA clone 4831435M15 3' similar to L08266 Mouse Facc mRNA, mRNA sequence. |
| 92718_at | 2.30 | 2310061N23Rik | AI158810 | ud38d09.r1 Soares_mammary_gland_NbMMG Mus musculus cDNA clone IMAGE:1448177 5' similar to SW:INI7_HUMAN P40305 INTERFERON-ALPHA INDUCED 11.5 KD PROTEIN ;, mRNA sequence.; RIKEN cDNA 2310061N23 gene |
| 96489_at | 2.30 |  | C81612 | C81612 Mouse 3.5-dpc blastocyst cDNA Mus musculus cDNA clone J0102C09 3' similar to putative transcription regulator {clone T2, repetitive, mRNA sequence. |
| 95911_at | 2.30 | 3010025C11Rik; 2900006B13Rik | AI585872 | vs53e08.x1 Stratagene mouse skin (#937313) Mus musculus cDNA clone IMAGE:1150022 3', mRNA sequence.; RIKEN cDNA 2900006B13 gene |
| 161323_f_at | 2.30 | Cad-pending | AV095547 | AV095547 Mus musculus C57BL/6J ES cell Mus musculus cDNA clone 2410008J01, mRNA sequence. |
| 101686_at | 2.30 | Zfp59 | D18410 | MUSGS01468 Mouse 3'-directed Mus musculus domesticus cDNA clone md1312 3', mRNA sequence.; zinc finger protein 59 |
| 162249_f_at | 2.30 | 2610200G18Rik | AV334573 | AV334573 RIKEN full-length enriched, adult male medulla oblongata Mus musculus cDNA clone 6330561N23 3', mRNA sequence. |
| 101009_at | 2.30 | Krt2-8; K8; Card2; EndoA; Krt-2.8 | X15662 | unnamed protein product; cytokeratin endo A; Mouse gene for cytokeratin endo A (no 8) 5' end. |
| 99021_at | 2.30 | Pmx; Prrx1; K-2; Pmx1; Prx1; mHox; A230024N07Rik | U03873 | Mus musculus homeobox (Pmx) mRNA, complete cds.; paired related homeobox 1 |
| 92450_at | 2.30 | Kcc1; Slc12a4; KCC1; RBCKCC1 | AF047339 | cation-chloride cotransporter; Mus musculus erythroid K:Cl cotransporter (Kcc1) mRNA, complete cds.; solute carrier family 12, member 4 |
| 160537_at | 2.30 | SULT-N; Sultn; ST1d1; SULT-N; 5033411P13Rik | AF026073 | Mus musculus amine N-sulfotransferase (SULT-N) mRNA, complete cds. |
| 160680_at | 2.24 | Cpeb1; mCPEB | Y08260 | M.musculus mRNA for CPEB protein.; cytoplasmic polyadenylation element binding protein 1 |
| 95386_at | 2.24 | AI181996 | AI847050 | UI-M-AP1-agn-a-03-0-UI.s1 NIH_BMAP_MST_N Mus musculus cDNA clone UI-M-AP1-agn-a-03-0-UI 3', mRNA sequence. |
| 100050_at | 2.24 | Idb1; Id1; D2Wsu140e | M31885 | helix-loop-helix protein (Id); Mouse helix-loop-helix DNA binding protein regulator (Id) mRNA, 3' end.; inhibitor of DNA binding 1 |
| 104712_at | 2.24 | Myc; Myc; Myc2; Nird; 84769; Niard; c-myc | L00039 | c-myc; Mus musculus c-myc (Myc) gene, exon 3 and complete cds. |
| 92441_at | 2.24 | Fap | Y10007 | M.musculus mRNA for fibroblast activation protein. |
| 103051_at | 2.24 | wdnm1; Expi; WDNM1 | X93037 | protease inhibitor homology; M.musculus mRNA for WDNM1 protein.; extracellular proteinase inhibitor |
| 104194_at | 2.24 | heph; Heph; sla; C130006F04Rik | AF082567 | similar to ceruloplasmin; mutant in the sla mice; highly expressed in small intestine; Mus musculus hephaestin (heph) mRNA, complete cds. |
| 93929_s_at | 2.24 | mrp/plf3; Mrpplf3; MRP-3; PLF-3; mrp/plf3 | X16009 | Mouse mrp/plf3 gene for mitogen regulated protein/proliferin (MRP/PLF), exon 1 (and joined CDS). |
| 162233_r_at | 2.24 | Slc38a2 | AV325582 | AV325582 RIKEN full-length enriched, adult male medulla oblongata Mus musculus cDNA clone 6330404L18 3', mRNA sequence. |
| 103907_at | 2.24 | Nedd4l | AW108492 | um31a04.x1 Sugano mouse kidney mkia Mus musculus cDNA clone IMAGE:2236110 3' similar to gb:D10714 Mouse mRNA of NEDD-4 gene, partial sequence (MOUSE);, mRNA sequence.; neural precursor cell expressed, developmentally down-regulated gene 4-like |
| 100676_at | 2.24 | Synj2; SJ2 | AF026123 | phosphatydilphosphate-5-phosphatase; Mus musculus synaptojanin 2 mRNA, partial cds. |
| 102694_at | 2.24 | bCEA; Psg16; bCEA; Cea11 | U34272 | pregnancy-specific glycoprotein subclass, carcinoembryonic antigen gene family.; Mus musculus brain carcinoembryonic antigen (bCEA) mRNA, complete cds.; pregnancy specific glycoprotein 16 |
| 99056_at | 2.24 | Pcbd | AW046590 | UI-M-BH1-alb-f-08-0-UI.s1 NIH_BMAP_M_S2 Mus musculus cDNA clone UI-M-BH1-alb-f-08-0-UI 3', mRNA sequence.; 6-pyruvoyl-tetrahydropterin synthase/dimerization cofactor of hepatocyte nuclear factor 1 alpha (TCF1) |
| 104157_at | 2.24 | C330018L13Rik | AW125643 | UI-M-BH2.2-aqi-a-09-0-UI.s1 NIH_BMAP_M_S3.2 Mus musculus cDNA clone UI-M-BH2.2-aqi-a-09-0-UI 3', mRNA sequence.; RIKEN cDNA C330018L13 gene |
| 98465_f_at | 2.24 | Ifi204 | M31419 | 204; Mouse 204 interferon-activatable protein mRNA, complete cds.; interferon activated gene 204 |
| 94939_at | 2.22 | CD53; Cd53; Ox-44 | X97227 | M.musculus mRNA for cell surface glycoprotein CD53.; CD53 antigen |
| 97257_at | 2.19 | Cgi-83-pending | AW122766 | UI-M-BH2.2-aot-h-10-0-UI.s1 NIH_BMAP_M_S3.2 Mus musculus cDNA clone UI-M-BH2.2-aot-h-10-0-UI 3', mRNA sequence.; Mus musculus 13 days embryo forelimb cDNA, RIKEN full-length enriched library, clone:5930433C01 product:unclassifiable, full insert sequence |
| 160993_at | 2.19 |  | AI854813 | UI-M-BH0-aka-h-03-0-UI.s1 NIH_BMAP_M_S1 Mus musculus cDNA clone UI-M-BH0-aka-h-03-0-UI 3', mRNA sequence. |
| 96931_at | 2.19 | Mrp63 | AW048260 | UI-M-BH1-aly-d-01-0-UI.s1 NIH_BMAP_M_S2 Mus musculus cDNA clone UI-M-BH1-aly-d-01-0-UI 3', mRNA sequence.; mitochondrial ribosomal protein 63 |
| 95892_at | 2.19 |  | AA189214 | mu50b07.r1 Soares mouse lymph node NbMLN Mus musculus cDNA clone IMAGE:642805 5', mRNA sequence.; Mus musculus transcribed sequences |
| 161372_f_at | 2.19 | Hnrpdl | AV240182 | AV240182 RIKEN full-length enriched, 10 day neonate skin Mus musculus cDNA clone 4732438P20 3', mRNA sequence. |
| 99914_at | 2.19 | Hed; Hand2; Hed; Th2; dHAND; Ehand2; Thing2 | U43715 | helix-loop-helix transcription factor; Mus musculus helix-loop-helix transcription factor (Hed) mRNA, partial cds.; heart and neural crest derivatives expressed transcript 2 |
| 161105_at | 2.19 | Txnl | AV296012 | AV296012 RIKEN full-length enriched, 8 days embryo Mus musculus cDNA clone 5730439B01 3', mRNA sequence.; Mus musculus transcribed sequences |
| 161551_f_at | 2.19 | Riok3 | AV247682 | AV247682 RIKEN full-length enriched, 0 day neonate head Mus musculus cDNA clone 4832437D20 3', mRNA sequence. |
| 98772_at | 2.19 | Cxcl5; LIX; GCP-2; Scyb5; Scyb6; ENA-78; AMCF-II | U27267 | LPS-induced C-X-C chemokine precursor; Mus musculus LPS-induced C-X-C chemokine LIX precursor, mRNA, complete cds.; chemokine (C-X-C motif) ligand 5 |
| 93397_at | 2.19 | Ccr2; CKR2; CCR2A; CCR2B; CKR2A; CKR2B; mJE-R; Cmkbr2; CC-CKR-2 | U56819 | human chemokine mcp-1 receptor homolog; Mus musculus mcp-1 receptor mRNA, complete cds.; chemokine (C-C) receptor 2 |
| 161200_i_at | 2.19 | Hspa8 | AV257761 | AV257761 RIKEN full-length enriched, adult male testis (DH10B) Mus musculus cDNA clone 4921539G10 3' similar to U27129 Mus musculus breast heat shock 73 protein (hsc73) mRNA, mRNA sequence. |
| 95725_at | 2.19 | 0610006H10Rik | AI314227 | uj35g09.x1 Sugano mouse kidney mkia Mus musculus cDNA clone IMAGE:1921984 3' similar to SW:ACY2_HUMAN P45381 ASPARTOACYLASE ;, mRNA sequence.; RIKEN cDNA 0610006H10 gene |
| 98726_at | 2.19 | PR; Pgr; PR; NR3C3; 9930019P03; 9930019P03Rik | M68915 | putative; Mouse progesterone receptor (PR) mRNA, complete cds. |
| 104292_at | 2.19 | Eya2 | U81603 | similar to Drosophila eyes absent gene; Mus musculus Eya2 homolog (Eya2) mRNA, complete cds.; eyes absent 2 homolog (Drosophila) |
| 97102_at | 2.19 | Yme1l1 | AA276948 | vc42f10.r1 Soares mouse 3NbMS Mus musculus cDNA clone IMAGE:777259 5', mRNA sequence.; YME1-like 1 (S. cerevisiae) |
| 101648_at | 2.19 | Foxd4; Fkh2; FREAC5 | X86368 | putative; M.musculus putative transcription factor. |
| 97754_at | 2.19 | G630024C07Rik | AI841211 | UI-M-AM0-adt-g-02-0-UI.s1 NIH_BMAP_MAM Mus musculus cDNA clone UI-M-AM0-adt-g-02-0-UI 3', mRNA sequence.; RIKEN cDNA G630024C07 gene |
| 92702_at | 2.19 | Astn1; GC14; mKIAA0289 | U48797 | neuronal migration protein; GC14; Mus musculus astrotactin mRNA, complete cds.; astrotactin 1 |
| 104072_at | 2.19 | Apcs; Sap | M23552 | amyloid P component precursor; Mouse serum amyloid P component mRNA, complete cds.; serum amyloid P-component |
| 93097_at | 2.19 | Arg1; AI; PGIF; Arg-1 | U51805 | Mus musculus arginase mRNA, complete cds.; arginase 1, liver |
| 103723_at | 2.19 | Il13ra1 | AA608387 | vo42b10.r1 Barstead mouse irradiated colon MPLRB7 Mus musculus cDNA clone IMAGE:1052539 5', mRNA sequence.; interleukin 13 receptor, alpha 1 |
| 97867_at | 2.19 | 11beta-HSD1A; Hsd11b1 | X83202 | M.musculus mRNA for 11beta-hydroxysteroid dehydrogenase/carbonyl reductase.; hydroxysteroid 11-beta dehydrogenase 1 |
| 160479_at | 2.19 | Cat; Cas1; Cs-1; Cas-1; 2210418N07 | M29394 | catalase; Mouse catalase mRNA, 3' end. |
| 102227_g_at | 2.19 | Cpxm2; Cpx2; CPX-2; 4632435C11Rik | AF017639 | N-terminal discoidin domain; Mus musculus carboxypeptidase X2 mRNA, complete cds.; carboxypeptidase X 2 (M14 family) |
| 162068_r_at | 2.19 | Emb | AV309223 | AV309223 RIKEN full-length enriched, 8 days embryo Mus musculus cDNA clone 5730577N08 3' similar to J03535 Mouse Ig-related glycoprotein-70 mRNA, mRNA sequence. |
| 162156_f_at | 2.19 | Srpx | AV021449 | AV021449 Mus musculus 18-day embryo C57BL/6J Mus musculus cDNA clone 1190023A21, mRNA sequence. |
| 162341_r_at | 2.19 | Akr1b3 | AV133992 | AV133992 Mus musculus C57BL/6J 10-11 day embryo Mus musculus cDNA clone 2810003K04, mRNA sequence. |
| 93927_f_at | 2.19 |  | L33954 | Mus musculus clone BPS3.23 germline Ig variable region heavy chain precursor gene, partial cds. |
| 103091_at | 2.19 | relB; Relb | M83380 | transcription factor; Mouse transcription factor relB mRNA, complete cds.; avian reticuloendotheliosis viral (v-rel) oncogene related B |
| 161971_r_at | 2.14 | Btk | AV227438 | AV227438 RIKEN full-length enriched, 14 days embryo liver Mus musculus cDNA clone 4430402G17 3' similar to L10627 Mus musculus, mRNA sequence. |
| 104664_at | 2.14 | Ches1 | AA725931 | vu85c07.r1 Stratagene mouse skin (#937313) Mus musculus cDNA clone IMAGE:1198188 5' similar to TR:O00409 O00409 CHECKPOINT SUPPRESSOR 1. ;, mRNA sequence.; checkpoint suppressor 1 |
| 98529_at | 2.14 | dPRP; Dtprp; DPRP; D/tPRP | AF011385 | Mus musculus decidual PRL-related protein (dPRP) mRNA, complete cds.; decidual/trophoblast prolactin-related protein |
| 161103_at | 2.14 | Lysal2 | AA250256 | mz58e06.r1 Soares mouse lymph node NbMLN Mus musculus cDNA clone IMAGE:717634 5', mRNA sequence.; lysosomal apyrase-like 2 |
| 100671_at | 2.14 | Ifna11; IFN-[a]11 | M68944 | Mouse alpha-interferon gene, complete cds. |
| 162382_f_at | 2.14 | Ccs | AV248506 | AV248506 RIKEN full-length enriched, 0 day neonate head Mus musculus cDNA clone 4833407A17 3', mRNA sequence. |
| 104361_at | 2.14 | LOC232337 | AI837260 | UI-M-AK0-add-h-08-0-UI.s1 NIH_BMAP_MHY Mus musculus cDNA clone UI-M-AK0-add-h-08-0-UI 3', mRNA sequence. |
| 161692_r_at | 2.14 | Cfh | AV228333 | AV228333 RIKEN full-length enriched, 14 days embryo liver Mus musculus cDNA clone 4432415F11 3' similar to M29010 Mouse complement factor H-related protein mRNA, mRNA sequence. |
| 104486_at | 2.14 | A2m | AI850558 | UI-M-BG1-aij-e-07-0-UI.s1 NIH_BMAP_MSC_N Mus musculus cDNA clone UI-M-BG1-aij-e-07-0-UI 3', mRNA sequence.; alpha-2-macroglobulin |
| 99620_at | 2.14 | Sfpq | AW060546 | UI-M-BH1-ann-g-04-0-UI.s1 NIH_BMAP_M_S2 Mus musculus cDNA clone UI-M-BH1-ann-g-04-0-UI 3', mRNA sequence.; Mus musculus transcribed sequence with moderate similarity to protein sp:P23246 (H.sapiens) SFPQ_HUMAN Splicing factor, proline-and glutamine-rich (Polypyrimidine tract-binding protein-associated splicing factor) (PTB-associated splicing factor) (PSF) (DNA-binding P52/P100 complex, 100 kDa subunit) |
| 103292_at | 2.14 | Stc1 | U47815 | Mus musculus stanniocalcin gene, complete cds. |
| 103710_at | 2.14 |  | AI037032 | ue17d02.x1 Sugano mouse embryo mewa Mus musculus cDNA clone IMAGE:1480611 3', mRNA sequence.; Mus musculus transcribed sequence with weak similarity to protein pir:T14757 (H.sapiens) T14757 hypothetical protein DKFZp572C163.1 - human (fragment) |
| 97013_f_at | 2.14 | Cyba | AW046124 | UI-M-BH1-alf-e-03-0-UI.s1 NIH_BMAP_M_S2 Mus musculus cDNA clone UI-M-BH1-alf-e-03-0-UI 3', mRNA sequence. |
| 94854_g_at | 2.14 | Gnb1; Gnb-1 | U29055 | G protein beta 1 subunit; Mus musculus G protein beta 36 subunit mRNA, complete cds.; guanine nucleotide binding protein, beta 1 |
| 104078_g_at | 2.14 | 1110049G11Rik | AW121992 | UI-M-BH2.3-aoj-f-11-0-UI.s1 NIH_BMAP_M_S3.3 Mus musculus cDNA clone UI-M-BH2.3-aoj-f-11-0-UI 3', mRNA sequence.; RIKEN cDNA 1110049G11 gene |
| 95964_at | 2.14 | 4932431F02Rik | C77404 | C77404 Mouse 3.5-dpc blastocyst cDNA Mus musculus cDNA clone J0030E06 3', mRNA sequence.; RIKEN cDNA 4932431F02 gene |
| 102769_f_at | 2.14 | C5D; Sc5d; A830037K02; A830073K23Rik | AB016248 | Mus musculus mRNA for sterol-C5-desaturase, complete cds.; sterol-C5-desaturase (fungal ERG3, delta-5-desaturase) homolog (S. cerevisae) |
| 161057_at | 2.14 | 2900086B20Rik | AI662504 | mt33a04.x1 Soares mouse 3NbMS Mus musculus cDNA clone IMAGE:622830 3', mRNA sequence.; Mus musculus adult male hippocampus cDNA, RIKEN full-length enriched library, clone:2900086B20 product:unknown EST, full insert sequence |
| 102908_at | 2.14 | mur42; Epb4.2 | U04055 | Erythrocyte membrane protein 4.2 is N-myristylated and binds to the cytoplasmic domain of the band 3 anion transport protein; Mus musculus C57Bl6 erythrocyte membrane protein band 4.2 (mur42) mRNA, complete cds.; erythrocyte protein band 4.2 |
| 100611_at | 2.14 | Lyzs; Lys; Lzm; Lzp; Lzm-s1 | M21050 | precursor; Mouse lysozyme M gene, exon 4. |
| 99058_at | 2.14 | HMGI-C; Hmga2; pg; Hmgic; pygmy; HMGI-C; 9430083A20Rik | X99915 | M.musculus HMGI-C gene, exon 1, and joined CDS. |
| 160095_at | 2.14 | rrg; Lox; TSC-160 | D10837 | Mus muculus rrg (ras recision gene) mRNA, partial sequence.; lysyl oxidase |
| 94723_at | 2.14 | NAT1; Nat1; Nat-1 | U37119 | Mus musculus arylamine N-acetyltransferase (NAT1) gene, complete cds. |
| 101305_at | 2.14 | Pou3f3; Brn1; Otf8; Brn-1; Skin1 | M88299 | Brain-1 class III POU-domain protein; Mouse brain-1 POU-domain protein, complete cds. |
| 100537_at | 2.14 | 2310016M24Rik | AI836443 | UI-M-AI0-aak-d-09-0-UI.s1 NIH_BMAP_MBS Mus musculus cDNA clone UI-M-AI0-aak-d-09-0-UI 3', mRNA sequence.; RIKEN cDNA 2310016M24 gene |
| 96539_at | 2.14 | 9330147J08Rik | AW212071 | uo81h11.x1 NCI_CGAP_Mam3 Mus musculus cDNA clone IMAGE:2648997 3', mRNA sequence. |
| 161921_f_at | 2.09 | Psmc5 | AV139898 | AV139898 Mus musculus C57BL/6J 10-11 day embryo Mus musculus cDNA clone 2810055C15, mRNA sequence. |
| 95621_at | 2.09 | 9030623C06Rik | AA606367 | vo47h02.r1 Barstead mouse irradiated colon MPLRB7 Mus musculus cDNA clone IMAGE:1053075 5', mRNA sequence.; RIKEN cDNA 9030623C06 gene |
| 98878_r_at | 2.09 | D030041N15Rik | AW050141 | UI-M-BH1-ano-e-10-0-UI.s1 NIH_BMAP_M_S2 Mus musculus cDNA clone UI-M-BH1-ano-e-10-0-UI 3', mRNA sequence.; RIKEN cDNA D030041N15 gene |
| 161553_i_at | 2.09 | A030009H04Rik | AV252253 | AV252253 RIKEN full-length enriched, 0 day neonate head Mus musculus cDNA clone 4833439A03 3', mRNA sequence. |
| 162075_r_at | 2.09 | Mpeg1 | AV281445 | AV281445 RIKEN full-length enriched, adult male testis (DH10B) Mus musculus cDNA clone 4933424J07 3' similar to L20315 Mus musculus MPS1 gene and mRNA, 3'end, mRNA sequence.; macrophage expressed gene 1 |
| 161991_at | 2.09 | Wbp2 | AV325341 | AV325341 RIKEN full-length enriched, adult male medulla oblongata Mus musculus cDNA clone 6330403C23 3' similar to U40826 Mus musculus WW-domain binding protein 2 mRNA, mRNA sequence. |
| 92425_at | 2.09 | Chd1l | AW122559 | UI-M-BH2.2-aox-e-05-0-UI.s1 NIH_BMAP_M_S3.2 Mus musculus cDNA clone UI-M-BH2.2-aox-e-05-0-UI 3', mRNA sequence.; chromodomain helicase DNA binding protein 1-like |
| 104425_at | 2.09 | Cipp; 5031439B21 | AF060539 | CIPP; Mus musculus channel interacting PDZ domain protein mRNA, complete cds.; channel-interacting PDZ domain protein |
| 102123_at | 2.09 | LAL; Lip1; Lal; Lipa; Lip-1 | Z31689 | M.musculus (C57 Black/6X CBA) LAL mRNA for lysosomal acid lipase.; lysosomal acid lipase 1 |
| 93542_at | 2.09 | Pter; Mpr56-1 | U28016 | Mus musculus parathion hydrolase (phosphotriesterase)-related protein mRNA, complete cds.; phosphotriesterase related |
| 160273_at | 2.09 | Zfp36l2 | AA960603 | vw64d05.s1 Soares_mammary_gland_NMLMG Mus musculus cDNA clone IMAGE:1248585 3', mRNA sequence.; Mus musculus transcribed sequence with weak similarity to protein ref:NP_500767.1 (C.elegans) F36H12.3.p [Caenorhabditis elegans] |
| 101162_at | 2.09 | myf-5; Myf5; B130010J22Rik | X56182 | M.musculus myf-5 mRNA.; myogenic factor 5 |
| 161914_s_at | 2.09 | Lsp1 | AV122642 | AV122642 Mus musculus C57BL/6J 10-day embryo Mus musculus cDNA clone 2610528F19, mRNA sequence.; Mus musculus, clone IMAGE:4949876, mRNA |
| 96433_at | 2.09 | Usp29 | AA673236 | vp49a08.r1 Knowles Solter mouse 2 cell Mus musculus cDNA clone IMAGE:1079990 3', mRNA sequence. |
| 103968_at | 2.09 | MID2; Mid2; FXY2; Trim1 | Y18881 | Mus musculus mRNA for MID2 protein.; midline 2 |
| 99874_at | 2.09 | Rap2b | AA959291 | ua14g10.r1 Soares_mammary_gland_NbMMG Mus musculus cDNA clone IMAGE:1346754 5', mRNA sequence.; Mus musculus 0 day neonate cerebellum cDNA, RIKEN full-length enriched library, clone:C230070N09 product:RAS-RELATED PROTEIN RAP-2B homolog [Homo sapiens], full insert sequence |
| 161680_r_at | 2.09 | Nr1h2 | AV160842 | AV160842 Mus musculus head C57BL/6J 12-day embryo Mus musculus cDNA clone 3010053P21, mRNA sequence. |
| 161663_f_at | 2.09 | 1810055D05Rik | AV130375 | AV130375 Mus musculus C57BL/6J 11-day embryo Mus musculus cDNA clone 2700077B01, mRNA sequence. |
| 94131_at | 2.09 | Xpo1 | AI451173 | mt67a02.x1 Soares mouse lymph node NbMLN Mus musculus cDNA clone IMAGE:634922 3', mRNA sequence.; exportin 1, CRM1 homolog (yeast) |
| 103770_at | 2.09 | 2310014L17Rik | AA794189 | vu67d02.r1 Stratagene mouse skin (#937313) Mus musculus cDNA clone IMAGE:1196451 5', mRNA sequence.; RIKEN cDNA 2310014L17 gene |
| 92474_at | 2.05 | PLD1; Pld1; Pld1a; Pld1b | AF083497 | Mus musculus phospholipase D1 (PLD1) gene, exons 27 and 28, complete sequence; and complete cds. |
| 94426_at | 2.05 | 6330575P11Rik | AI851052 | UI-M-BH0-ajv-g-02-0-UI.s1 NIH_BMAP_M_S1 Mus musculus cDNA clone UI-M-BH0-ajv-g-02-0-UI 3', mRNA sequence.; RIKEN cDNA 6330575P11 gene |
| 99126_at | 2.05 | Xist; A430022B11 | L04961 | Mouse nuclear-localized inactive X-specific transcript (Xist) mRNA.; Mus musculus 0 day neonate thymus cDNA, RIKEN full-length enriched library, clone:A430022B11 product:inactive X specific transcripts, full insert sequence |
| 98825_at | 2.05 | Kcne1; Isk; MinK | X60457 | M.musculus mRNA for K Channel.; potassium voltage-gated channel, Isk-related subfamily, member 1 |
| 98345_at | 2.05 | AA536748 | AA674593 | vm73h01.s1 Knowles Solter mouse 2 cell Mus musculus cDNA clone IMAGE:1003921 5', mRNA sequence.; Mus musculus transcribed sequences |
| 102791_at | 2.05 | Lmp7; Psmb8; Lmp7; Lmp-7 | U22033 | Lmp7k, s, f allele; Mus musculus 20S proteasome subunit Lmp7 (Lmp7k, s, f allele) mRNA, complete cds.; proteosome (prosome, macropain) subunit, beta type 8 (large multifunctional protease 7) |
| 99958_at | 2.05 | Mcpt2; Mcp-2; MMCP-2 | J05177 | Mouse mast cell protease-2 (MMCP-2) mRNA, complete cds.; mast cell protease 2 |
| 93849_at | 2.05 | mTPK1; Tpk1 | AB027568 | Mus musculus mTPK1 mRNA for thiamin pyrophosphokinase, complete cds. |
| 161001_at | 2.05 | AA407270 | AV367683 | AV367683 RIKEN full-length enriched, 16 days embryo lung Mus musculus cDNA clone 8430436K21 3', mRNA sequence.; Mus musculus 16 days embryo head cDNA, RIKEN full-length enriched library, clone:C130030N11 product:unclassifiable, full insert sequence |
| 93332_at | 2.05 | Cd36; FAT; GPIV; Scarb3 | L23108 | Mus musculus CD36 antigen mRNA, complete cds. |
| 94108_at | 2.05 | Pik3c2g | AB008791 | Mus musculus mRNA for Phosphoinositide 3-Kinase-C2gamma, complete cds.; phosphatidylinositol 3-kinase, C2 domain containing, gamma polypeptide |
| 93837_at | 2.05 | Kng | AI786089 | uj58h09.y1 Sugano mouse liver mlia Mus musculus cDNA clone IMAGE:1924193 5' similar to gb:K02566 KININOGEN, LMW PRECURSOR (HUMAN);, mRNA sequence.; kininogen |
| 101189_at | 2.05 | DP5; Bid3; DP5; Hrk; harakiri | D83698 | Mus musculus DP5 mRNA for neuronal death protein, complete cds.; BH3 interacting (with BCL2 family) domain, apoptosis agonist |
| 161055_r_at | 2.05 | 6330409N04Rik | AW047521 | UI-M-BH1-ama-d-01-0-UI.s1 NIH_BMAP_M_S2 Mus musculus cDNA clone UI-M-BH1-ama-d-01-0-UI 3', mRNA sequence.; RIKEN cDNA 6330409N04 gene |
| 161096_at | 2.05 |  | AA209597 | mw75e05.r1 Soares mouse NML Mus musculus cDNA clone IMAGE:676544 5', mRNA sequence.; Mus musculus mRNA similar to zinc finger protein (cDNA clone MGC:51479 IMAGE:4012958), complete cds |
| 162368_r_at | 2.05 | 1110033J02Rik | AV239112 | AV239112 RIKEN full-length enriched, 10 day neonate skin Mus musculus cDNA clone 4732431L21 3', mRNA sequence. |
| 101646_at | 2.05 | GPI1; Pigq; Gpi1; Gpih; Gpi1h; Gpi1p | AF030178 | Mus musculus N-acetylglucosaminyl transferase component Gpi1 (GPI1) mRNA, complete cds.; phosphatidylinositol glycan, class Q |
| 104484_at | 2.05 | STa2; Sth2; mSTa2 | L27121 | putative; Mus musculus (10-1) hydroxysteroid sulfotransferase (mSTa2) mRNA, complete cds.; sulfotransferase, hydroxysteroid preferring 2 |
| 100688_at | 2.05 | Crybb2; Aey2; Phil; Cryb-2 | M60559 | Mus musculus Swiss Webster beta-B2 crystallin mRNA, complete cds.; crystallin, beta B2 |
| 97265_at | 2.05 | 1810013D10Rik | AI835060 | UI-M-AI1-afn-e-12-0-UI.s1 NIH_BMAP_MBS_N Mus musculus cDNA clone UI-M-AI1-afn-e-12-0-UI 3', mRNA sequence.; RIKEN cDNA 1810013D10 gene |
| 93188_at | 2.05 | dkk-3; Dkk3 | AJ243964 | Mus musculus mRNA for dickkopf-3 (dkk-3 gene).; dickkopf homolog 3 (Xenopus laevis) |
| 100499_at | 2.05 | Stx3 | D29797 | Mouse mRNA for syntaxin 3A, complete cds. |
| 161828_r_at | 2.05 | AA408650 | AV022820 | AV022820 Mus musculus 18-day embryo C57BL/6J Mus musculus cDNA clone 1190029K04, mRNA sequence. |
| 101637_at | 2.05 | Cea10; Ceacam10; Bgp3; Cea10 | L38422 | putative; Mus musculus (clone lambda Cea10a) carcinoembryonic antigen (Cea10) mRNA, complete cds.; CEA-related cell adhesion molecule 10 |
| 100596_at | 2.00 | Selenbp1; Lp56; Lpsb; MGC18519 | M32032 | Selenium-binding liver protein; Mus musculus Selenium-binding liver protein mRNA.; selenium binding protein 1 |
| 99327_at | 2.00 | Prss19; BSP1; Nrpn | D30785 | nervous system; Mouse mRNA for neuropsin, complete cds.; protease, serine, 19 (neuropsin) |
| 162139_r_at | 2.00 | 2310044H10Rik | AV344835 | AV344835 RIKEN full-length enriched, adult male olfactory bulb Mus musculus cDNA clone 6430547N19 3', mRNA sequence. |
| 93037_i_at | 2.00 | Lipo 1; Anxa1; Lpc1; Anx-1; Lpc-1; Anx-A1 | M69260 | Mouse lipocortin I gene, exon 13. |
| 93334_at | 2.00 | VLCAD; Acadvl; VLCAD | Y11770 | Mus musculus VLCAD gene. |
| 101790_f_at | 2.00 | Ifna4; Ifa4 | X01973 | Mouse gene for interferon alpha 4 (Mu IFN-alpha 4). |
| 92475_g_at | 2.00 | PLD1; Pld1; Pld1a; Pld1b | AF083497 | Mus musculus phospholipase D1 (PLD1) gene, exons 27 and 28, complete sequence; and complete cds. |
| 99613_at | 2.00 | Mut | X51941 | unnamed protein product; precursor polypeptide (AA -30 to 718); Mouse mRNA for methylmalonyl CoA mutase (EC 5.4.99.2).; methylmalonyl-Coenzyme A mutase |
| 93445_at | 2.00 | Cd5l; Api6; AAC-11; Pdp 1/6; Sp-alpha | AF011428 | Mus musculus CT-2 mRNA, complete cds.; CD5 antigen-like |
| 99806_at | 2.00 | Npr3; lgj; stri; NPR-C | D78175 | Mouse mRNA for ANP-clearance receptor, complete cds.; natriuretic peptide receptor 3 |
| 93430_at | 2.00 | Cmkor1; Rdc1 | AF000236 | seven transmembrane receptor; Mus musculus RDC1 orphan chemokine receptor mRNA, complete cds.; chemokine orphan receptor 1 |
| 92331_at | 2.00 | CD10/NEP; Mme; NEP; CD10; CALLA; 6030454K05Rik | M81591 | putative; Murine CD10 neutral endopeptidase 24.11 (CD10/NEP) mRNA, putative cds.; membrane metallo endopeptidase |
| 160953_at | 2.00 | Cacna1h; Cav3.2; MNCb-1209; alpha13.2 | AF051947 | low voltage-activated; alpha 1H; similar to product encoded by Genbank Accession Number W76774; Mus musculus T-type calcium channel alpha-1 subunit mRNA, partial cds.; calcium channel, voltage-dependent, T type, alpha 1H subunit |
| 160684_at | 2.00 | 8430410A17Rik | AA930519 | vo45f01.r1 Barstead mouse irradiated colon MPLRB7 Mus musculus cDNA clone IMAGE:1052857 5', mRNA sequence.; RIKEN cDNA 8430410A17 gene |
| 161022_at | 2.00 | 2810425M01Rik | AV322780 | AV322780 RIKEN full-length enriched, 11 days embryo head Mus musculus cDNA clone 6230401N03 3', mRNA sequence. |
| 98457_at | 2.00 | Slc4a4; NBC | AF020195 | Mus musculus pancreas sodium bicarbonate cotransporter mRNA, complete cds.; solute carrier family 4 (anion exchanger), member 4 |
| 96729_at | 2.00 | BC010348 | AI848377 | UI-M-AH1-ago-f-05-0-UI.s1 NIH_BMAP_MCE_N Mus musculus cDNA clone UI-M-AH1-ago-f-05-0-UI 3', mRNA sequence.; Mus musculus 10, 11 days embryo whole body cDNA, RIKEN full-length enriched library, clone:2810011I19 product:unknown EST, full insert sequence |
| 97108_at | 2.00 | Recql5 | AW049927 | UI-M-BH1-ami-h-11-0-UI.s1 NIH_BMAP_M_S2 Mus musculus cDNA clone UI-M-BH1-ami-h-11-0-UI 3', mRNA sequence. |
| 160243_r_at | 2.00 | Myh8; Myhsp; Myhs-p; MyHC-pn; Myh4 | M12289 | myosin heavy chain; Mouse perinatal skeletal myosin heavy chain mRNA, 3' end.; myosin, heavy polypeptide 4, skeletal muscle |
| 104156_r_at | 2.00 | Atf3; LRG-21 | U19118 | leucine zipper protein; Mus musculus transcription factor LRG-21 mRNA, complete cds.; activating transcription factor 3 |
| 98131_at | 2.00 | Cryz; Sez9 | D78646 | Mouse mRNA for zeta-crystallin/quinone reductase, partial cds.; crystallin, zeta |
| 104370_s_at | 2.00 | KER2; Krt2-6a; MK6a; mK6[a]; Krt2-6c; 2310016L08Rik | K02108 | Mouse keratin (epidermal) intermediate filament subunit II, mRNA.; keratin complex 2, basic, gene 6a |
| 98835_at | 2.00 | sema3A; Sema3a; SemD; SEMA1; Semad; coll-1; Hsema-I | D85028 | Mus musculus sema3A mRNA for semaphorin 3A, complete cds.; sema domain, immunoglobulin domain (Ig), short basic domain, secreted, (semaphorin) 3A |
| 161746_i_at | 2.00 | 1110064N10Rik | AV320590 | AV320590 RIKEN full-length enriched, 13 days embryo male testis Mus musculus cDNA clone 6030424M03 3', mRNA sequence. |
| 161821_f_at | 2.00 | Entpd2 | AV378405 | AV378405 RIKEN full-length enriched, adult male cecum Mus musculus cDNA clone 9130414M04 3' similar to AF042811 Mus musculus ecto-ATPase mRNA, mRNA sequence. |
| 94803_at | 2.00 | PBX1b; Pbx1; Pbx-1; D230003C07Rik | AF020197 | Homeodomain protein; Transcription factor; Mus musculus transcription factor PBX1b (PBX1b) mRNA, complete cds.; pre B-cell leukemia transcription factor 1 |
| 94948_at | 2.00 | Zrp1; Trip6 | AF097511 | mZRP-1; Mus musculus zyxin related protein-1 (Zrp1) mRNA, complete cds.; thyroid hormone receptor interactor 6 |
| 95426_at | 2.00 | Echs1 | AW048512 | UI-M-BH1-alj-h-09-0-UI.s1 NIH_BMAP_M_S2 Mus musculus cDNA clone UI-M-BH1-alj-h-09-0-UI 3', mRNA sequence.; enoyl Coenzyme A hydratase, short chain, 1, mitochondrial |
| 95455_at | 2.00 | Dnpep | AF005051 | similar to yeast aminopeptidase 1, specific for N-terminal acidic amino acids of peptides, Swiss-Prot Accession Number P14904; Mus musculus aspartyl aminopeptidase mRNA, complete cds. |
| 161604_r_at | 2.00 | Kifap3 | AV308714 | AV308714 RIKEN full-length enriched, 8 days embryo Mus musculus cDNA clone 5730569G17 3' similar to D50367 House mouse; Musculus domesticus mRNA for KAP3B, mRNA sequence.; kinesin-associated protein 3 |
| 161587_at | 2.00 | Hrsp12 | AV321289 | AV321289 RIKEN full-length enriched, 13 days embryo male testis Mus musculus cDNA clone 6030432D09 3' similar to D49363 Rat mRNA for perchrolic acid soluble protein, mRNA sequence. |
| 103890_at | 2.00 | AW538196 | AW050153 | UI-M-BH1-ano-g-01-0-UI.s1 NIH_BMAP_M_S2 Mus musculus cDNA clone UI-M-BH1-ano-g-01-0-UI 3', mRNA sequence. |
