# Supplementary material for: Microarray analysis of androgen-regulated gene expression in testis: the use of the androgen-binding protein (ABP)-transgenic mouse as a model
Source: Reprod Biol Endocrinol. 2005 Dec 9;3:70. doi: 10.1186/1477-7827-3-70 (PMC1327675; doi:10.1186/1477-7827-3-70)
Supplement: Additional File 2 — This file is a Table listing all of the 198 genes whose expression was down-regulated by a factor of 2 or more in the testes of ABP-transgenic mice as compared to controls. [file 1477-7827-3-70-S2.doc]

| **AffyID** | **Fold Decrease** | **Common Name** | **GenBank ID** | **Description** |
| --- | --- | --- | --- | --- |
| 161863_r_at | 13.00 | Cd3d | AV067753 | AV067753 Mus musculus small intestine C57BL/6J adult Mus musculus cDNA clone 2010208N07, mRNA sequence.; CD3 antigen, delta polypeptide |
| 162217_r_at | 11.06 | Fdps | AV090583 | AV090583 Mus musculus tongue C57BL/6J adult Mus musculus cDNA clone 2310050P21, mRNA sequence. |
| 102725_at | 9.40 | KVB1; Kcnab1; Akr8a8; mkv(beta)1 | AF033003 | similar to K+ channel beta subunit of mouse fibroblast, GenBank Accession Number X97281 and of rat nervous tissue, GenBank Accession Number X70662; Mus musculus K+ channel beta-1 subunit (KVB1) mRNA, complete cds.; potassium voltage-gated channel, shaker-related subfamily, beta member 1 |
| 162378_r_at | 7.46 | 8430437G11Rik | AV245223 | AV245223 RIKEN full-length enriched, 0 day neonate head Mus musculus cDNA clone 4831436O12 3', mRNA sequence. |
| 161637_f_at | 6.28 | Klk5 | AV053185 | AV053185 Mus musculus pancreas C57BL/6J adult Mus musculus cDNA clone 1810025M09, mRNA sequence. |
| 161540_r_at | 6.06 | 2610318G18Rik | AV350451 | AV350451 RIKEN full-length enriched, adult male cerebellum Mus musculus cDNA clone 6530435A05 3', mRNA sequence. |
| 162135_r_at | 4.59 | Slc38a2 | AV379830 | AV379830 RIKEN full-length enriched, adult male epididymis Mus musculus cDNA clone 9230014M14 3', mRNA sequence. |
| 161290_r_at | 4.49 | Brd7 | AV302662 | AV302662 RIKEN full-length enriched, 8 days embryo Mus musculus cDNA clone 5730504P12 3' similar to AF084259 Mus musculus bromodomain-containing protein BP75 mRNA, mRNA sequence. |
| 99198_at | 4.09 | 3A | K02060 | Mus musculus ribosomal protein L32-3A (3A) gene, complete cds. |
| 162170_r_at | 4.09 | Fbn1 | AV363605 | AV363605 RIKEN full-length enriched, 15 days embryo male testis Mus musculus cDNA clone 8030428I21 3' similar to U22493 Mus musculus fibrillin-1 (Fbn1) mRNA, mRNA sequence. |
| 102575_at | 4.00 | AGP-3; Orm3; Agp-3; Orm-3 | S38219 | single-chain plasma peptide; This sequence comes from Fig. 2; conceptual translation presented here differs from translation in publication; Mus musculus alpha 1-acid glycoprotein-3 gene, complete cds. |
| 102705_at | 3.73 | Il2; Il-2 | K02292 | interleukin 2 prepeptide; Mouse interleukin-2 mRNA, complete cds. |
| 96488_at | 3.73 | Jak2 | AV246464 | AV246464 RIKEN full-length enriched, 0 day neonate head Mus musculus cDNA clone 4832410F01 3', mRNA sequence.; Janus kinase 2 |
| 162476_r_at | 3.65 | Rpl8 | AV092840 | AV092840 Mus musculus tongue C57BL/6J adult Mus musculus cDNA clone 2310075N19, mRNA sequence. |
| 161940_r_at | 3.65 | Rnf103 | AV336118 | AV336118 RIKEN full-length enriched, adult male medulla oblongata Mus musculus cDNA clone 6330582C11 3' similar to D76445 Mouse mkf-1 mRNA, mRNA sequence. |
| 98023_r_at | 3.65 | Plfr; PRP; PLF-RP | K03237 | unnamed protein product; PRP precursor (aa -25 to 219); Mouse mRNA for proliferin-related protein (PRP). |
| 95882_at | 3.56 |  | AI592102 | mr23c08.y1 Soares mouse 3NbMS Mus musculus cDNA clone IMAGE:598286 5', mRNA sequence.; Mus musculus transcribed sequences |
| 104329_at | 3.48 | Smarcf1 | AI842326 | UI-M-AM1-afz-b-09-0-UI.s1 NIH_BMAP_MAM_N Mus musculus cDNA clone UI-M-AM1-afz-b-09-0-UI 3', mRNA sequence.; SWI/SNF related, matrix associated, actin dependent regulator of chromatin, subfamily f, member 1 |
| 101201_r_at | 3.48 |  | T25605 | EST00645 Unequalized cDNA library from Ltk- cultured cells Mus musculus cDNA clone B0001H07 3', mRNA sequence. |
| 100722_r_at | 3.48 | LOC56304 | U10410 | Mus musculus recombinant antineuraminidase single chain Ig VH and VL domains mRNA, complete cds. |
| 162291_r_at | 3.48 | 8430437G11Rik | AV377018 | AV377018 RIKEN full-length enriched, adult male cecum Mus musculus cDNA clone 9130219P05 3', mRNA sequence. |
| 103577_at | 3.40 | Pfkfb3 | AI326331 | ml59b10.x1 Stratagene mouse testis (#937308) Mus musculus cDNA clone IMAGE:516283 3' similar to TR:O35557 O35557 FRUCTOSE-6-PHOSPHATE 2-KINASE/FRUCTOSE-2,6-BISPHOSPHATASE. [1] ;, mRNA sequence.; 6-phosphofructo-2-kinase/fructose-2,6-biphosphatase 3 |
| 93167_f_at | 3.40 | Olr1 | AA104818 | mo58d05.r1 Life Tech mouse embryo 8 5dpc 10664019 Mus musculus cDNA clone IMAGE:557769 5', mRNA sequence.; Mus musculus transcribed sequences |
| 100414_s_at | 3.32 | Mpo | X15313 | unnamed protein product; prepromyeloperoxidase; Mouse MPO mRNA for myeloperoxidase (EC 1.11.7). |
| 161866_at | 3.32 |  | AV341298 | AV341298 RIKEN full-length enriched, adult male olfactory bulb Mus musculus cDNA clone 6430520H08 3' similar to X65138 M.musculus mRNA for tyrosine kinase, mRNA sequence. |
| 100319_at | 3.32 | Il10; CSIF; Il-10 | M37897 | interleukin 10 precursor; Mouse interleukin 10 mRNA, complete cds. |
| 102338_at | 3.17 | PDE9A*1; Pde9a; PDE9A1 | AF031147 | family 9 phosphodiesterase; PDE9A1; Mus musculus cGMP phosphodiesterase (PDE9A*1) mRNA, complete cds.; phosphodiesterase 9A |
| 162193_f_at | 3.17 | Itgb7 | AV371872 | AV371872 RIKEN full-length enriched, adult male colon Mus musculus cDNA clone 9030421D19 3' similar to M95632 Mouse integrin beta-7 subunit mRNA, mRNA sequence.; integrin beta 7 |
| 161276_i_at | 3.17 | Sox3 | AV324911 | AV324911 RIKEN full-length enriched, 11 days embryo head Mus musculus cDNA clone 6230429O11 3', mRNA sequence. |
| 161820_f_at | 3.17 | Gata2 | AV377670 | AV377670 RIKEN full-length enriched, adult male cecum Mus musculus cDNA clone 9130233I18 3' similar to AB000096 Mouse mRNA for GATA-2 protein, mRNA sequence. |
| 99367_at | 3.17 | Mapre1 | AA434661 | ve16g03.r1 Soares mouse NbMH Mus musculus cDNA clone IMAGE:818356 5', mRNA sequence.; microtubule-associated protein, RP/EB family, member 1 |
| 104474_s_at | 3.10 | Oprl; KOR3; ORL1; XOR1; morc; LC132; MOR-C | AF043276 | KOR3A; alternatively spliced; Mus musculus kappa3 related opioid receptor isoform A mRNA, complete cds.; opioid receptor-like |
| 102614_at | 3.03 | Prox1 | AF061576 | PROX1; Mus musculus prospero-related homeobox 1 (Prox1) mRNA, complete cds. |
| 93197_at | 3.03 |  | AF109905 | Cluster Incl AF109905:Mus musculus major histocompatibility locus class III regions Hsc70t gene, partial cds; smRNP, G7A, NG23, MutS homolog, CLCP, NG24, NG25, and NG26 genes, complete cds; and unknown genes /cds=(0,380) /gb=AF109905 /gi=3986751 /ug=Mm.56 |
| 103977_at | 2.96 | F10; fX; Cf10 | AF087644 | serine protease; Mus musculus coagulation factor X precursor, mRNA, complete cds. |
| 97954_at | 2.96 | tyrosinase-related protein-1 gene; Tyrp1; b; isa; TRP-1; brown | X59513 | Mouse 5' end of TRP1 gene for tyrosinase-related protein-1. |
| 100505_at | 2.96 | Rit2; Rin; RIBA; Roc2 | U71202 | expressed only in neurons; binds GTP and is localized to the plasma membrane in vitro; binds calmodulin; similar to Rhizomucor racemosus ras-like protein 2, Swiss-Prot Accession Number P22279; Mus musculus rin mRNA, complete cds.; Ras-like without CAAX 2 |
| 161089_r_at | 2.96 | Akap8 | AV367756 | AV367756 RIKEN full-length enriched, 16 days embryo lung Mus musculus cDNA clone 8430437G24 3' similar to U01914 Rattus norvegicus AKAP95 mRNA, mRNA sequence. |
| 94741_at | 2.93 | Tesp2 | AB008911 | Mus musculus mRNA for TESP2, complete cds.; testicular serine protease 2 |
| AFFX-MurIL2_at | 2.89 |  |  | M16762 Mouse interleukin 2 (IL-2) gene, exon 4 |
| 161069_at | 2.89 | Neurog1 | AI323358 | mi30g10.x1 Soares mouse embryo NbME13.5 14.5 Mus musculus cDNA clone IMAGE:465090 3', mRNA sequence. |
| 95797_f_at | 2.83 | VR1; V2r1 | AF011411 | Mus musculus putative pheromone receptor (VR1) mRNA, complete cds.; Mus musculus similar to putative pheromone receptor (LOC236461), mRNA |
| 97679_at | 2.83 | PMUR10F; Tlx2; Enx; NCX; Ncx1; Tlx1l1; Tlx1l2; Hox11l1; Hox11L.1 | M75953 | homologue of human HOX-11; putative; M.musculus homeobox containing protein (PMUR10F) mRNA, complete cds.; T-cell leukemia, homeobox 2 |
| 160474_at | 2.83 | 2610511E03Rik | AW046278 | UI-M-BH1-ala-g-04-0-UI.s1 NIH_BMAP_M_S2 Mus musculus cDNA clone UI-M-BH1-ala-g-04-0-UI 3', mRNA sequence.; RIKEN cDNA 2610511E03 gene |
| 160689_r_at | 2.83 | 9130005N23Rik | AW121848 | UI-M-BH2.3-aoi-b-01-0-UI.s1 NIH_BMAP_M_S3.3 Mus musculus cDNA clone UI-M-BH2.3-aoi-b-01-0-UI 3', mRNA sequence.; Mus musculus adult male cecum cDNA, RIKEN full-length enriched library, clone:9130005N23 product:similar to TNF RECEPTOR TYPE 1 ASSOCIATED DEATH DOMAIN PROTEIN, full insert sequence |
| 95334_at | 2.76 | trkC; Ntrk3; TrkC | AF035400 | mTrkC NC2; non-catalytic NT-3 receptor; Mus musculus neurotrophin-3 receptor non-catalytic isoform 2 (trkC) mRNA, complete cds.; neurotrophic tyrosine kinase, receptor, type 3 |
| 99909_at | 2.76 | Trp6; Trpc6; Trrp6; mTRP6; mtrp6 | U49069 | mTRP6; putative capacitative calcium entry channel; TRP (transient receptor potential) homolog; Mus musculus calcium entry channel (Trp6) mRNA, complete cds.; transient receptor potential cation channel, subfamily C, member 6 |
| 161202_r_at | 2.76 | Pdgfra | AV242260 | AV242260 RIKEN full-length enriched, 0 day neonate head Mus musculus cDNA clone 4831406D03 3' similar to M84607 Mus musculus PDGF-alpha-receptor (PDGF-alpha-R) mRNA, mRNA sequence. |
| 102898_at | 2.76 | HGF; Hgf; NK1; NK2; HGF/SF; SF/HGF; C230052L06Rik | X72307 | M.musculus mRNA for hepatocyte growth factor. |
| 101087_r_at | 2.73 | Cnbp | X63866 | Mus musculus mRNA for cellular nucleic acid binding protein. |
| 161961_at | 2.70 | Rpl19 | AV366598 | AV366598 RIKEN full-length enriched, 16 days embryo lung Mus musculus cDNA clone 8430421M22 3' similar to M62952 Mus musculus ribosomal protein L19, mRNA sequence. |
| 92414_at | 2.70 | Adam12; M[a]; Mltna | D50411 | Mus musculus mRNA for meltrin alpha, complete cds.; a disintegrin and metalloproteinase domain 12 (meltrin alpha) |
| 92843_r_at | 2.70 | E030015G23Rik | AW046013 | UI-M-BH1-alc-f-09-0-UI.s1 NIH_BMAP_M_S2 Mus musculus cDNA clone UI-M-BH1-alc-f-09-0-UI 3', mRNA sequence.; RIKEN cDNA E030015G23 gene |
| 99812_at | 2.70 | canp3; Capn3; Lp82; Capa3; Capa-3 | X92523 | M.musculus mRNA for skeletal muscle-specific calpain.; calpain 3 |
| 161754_f_at | 2.70 | Glb1 | AV292258 | AV292258 RIKEN full-length enriched, 6 days neonate head Mus musculus cDNA clone 5430419M10 3' similar to M57734 Mouse beta-galactosidase (BGAL) gene, mRNA sequence. |
| 95798_f_at | 2.70 | VR12; V2r12; V2r9 | AF011422 | truncated form; Mus musculus putative pheromone receptor (VR12) mRNA, complete cds.; vomeronasal 2, receptor, 9 |
| 100391_at | 2.70 | Mapk8; JNK; JNK1; Prkm8; SAPK1 | AB005663 | Mus musculus mRNA for JNK1, complete cds.; mitogen activated protein kinase 8 |
| 94129_at | 2.64 |  | AA164101 | mr23g07.r1 Soares mouse 3NbMS Mus musculus cDNA clone IMAGE:598332 5', mRNA sequence.; Mus musculus transcribed sequence with weak similarity to protein ref:NP_286085.1 (E. coli) beta-D-galactosidase [Escherichia coli O157:H7 EDL933] |
| 104336_at | 2.64 | 5230400M11Rik | AI843273 | UI-M-AO1-aei-d-01-0-UI.s1 NIH_BMAP_MPG_N Mus musculus cDNA clone UI-M-AO1-aei-d-01-0-UI 3', mRNA sequence.; RIKEN cDNA 5230400M11 gene |
| 99834_at | 2.64 | NRG3; Nrg3 | AF010130 | Mus musculus neuregulin-3 (NRG3) mRNA, complete cds.; neuregulin 3 |
| 100496_at | 2.64 | PAM; Pam | U79523 | Mus musculus peptidylglycine alpha-amidating monooxygenase (PAM) mRNA, complete cds. |
| 95325_at | 2.58 | Pscd1; CLM1; CTH-1; CYTIP | AF051337 | Mus musculus cytohesin-1 mRNA, complete cds.; pleckstrin homology, Sec7 and coiled-coil domains 1 |
| 96787_at | 2.58 | Serpina10 | AA880891 | vx33c07.r1 Stratagene mouse lung 937302 Mus musculus cDNA clone IMAGE:1277004 5' similar to TR:Q62975 Q62975 RASP1 PRECURSOR. ;, mRNA sequence. |
| 104436_at | 2.52 | Il1f5; IL-1H3; IL1HY1; FIL1delta | AJ250429 | Mus musculus mRNA for IL-1L1 protein.; interleukin 1 family, member 5 (delta) |
| 95347_at | 2.52 | Myt1; NZF-2a; NZF-2b | AF004294 | Myt1; Mus musculus myelin transcription factor 1 mRNA, complete cds. |
| 161764_r_at | 2.52 | Stac | AV334462 | AV334462 RIKEN full-length enriched, adult male medulla oblongata Mus musculus cDNA clone 6330557K16 3' similar to D86639 Mouse mRNA for stac, mRNA sequence. |
| 94353_at | 2.52 | Eif4ebp2; 4E-BP2; PHAS-II | U75530 | eIF-4E binding protein; also known as 4EBP-2; Mus musculus PHAS-II mRNA, complete cds.; eukaryotic translation initiation factor 4E binding protein 2 |
| 100670_at | 2.52 | Scn9a | L42338 | Mus musculus sodium channel 25 mRNA, complete cds.; sodium channel, voltage-gated, type IX, alpha polypeptide |
| 161804_r_at | 2.52 | 2010012D11Rik | AV369984 | AV369984 RIKEN full-length enriched, adult male colon Mus musculus cDNA clone 9030204I01 3', mRNA sequence. |
| 101188_at | 2.46 | GIRK1; Kcnj3; GIRK1; Kcnf3; Kir3.1 | D45022 | Mouse GIRK1 mRNA for G protein coupled inward rectifying potassium channel 1, complete cds.; potassium inwardly-rectifying channel, subfamily J, member 3 |
| 94098_at | 2.46 | Hcrt; Hcrt; PPOX | AF019566 | precursor of hypocretin neuropeptides; Mus musculus preprohypocretin (Hcrt) mRNA, complete cds. |
| 104325_at | 2.46 | 1110025G12Rik | AI461631 | uc42e08.x1 Soares_mammary_gland_NMLMG Mus musculus cDNA clone IMAGE:1400678 3', mRNA sequence.; RIKEN cDNA 1110025G12 gene |
| 104393_at | 2.46 | 2810453I06Rik | AI843571 | UI-M-AO1-aen-d-09-0-UI.s1 NIH_BMAP_MPG_N Mus musculus cDNA clone UI-M-AO1-aen-d-09-0-UI 3', mRNA sequence. |
| 161043_r_at | 2.46 | Txnrd3-pending | AV277568 | AV277568 RIKEN full-length enriched, adult male testis (DH10B) Mus musculus cDNA clone 4932443C12 3', mRNA sequence. |
| 161957_r_at | 2.46 | 2610034N03Rik | AV364085 | AV364085 RIKEN full-length enriched, 15 days embryo male testis Mus musculus cDNA clone 8030438G22 3', mRNA sequence. |
| 103066_at | 2.46 | Tyki; TDKI; 1200004E04Rik | L32973 | This ORF is capable of encoding 432 aa which is similar to thymidylate kinases especially at two domains: the p-loop or catalytic site and the substrate binding site; ORF; Mouse thymidylate kinase homologue mRNA, complete cds. |
| 98467_at | 2.46 | itih-4; Itih4; Itih-4 | AF023919 | Mus musculus PK-120 precursor (itih-4) mRNA, complete cds.; inter alpha-trypsin inhibitor, heavy chain 4 |
| 94271_at | 2.46 | Dmp1; Dmtf1; Dimp; Dmp1 | U70017 | Description: Myb-like transcription factor; Mus musculus cyclin D-interacting myb-like protein (Dmp1) mRNA, complete cds.; cyclin D binding myb-like transcription factor 1 |
| 99922_at | 2.46 | NHE-1; Slc9a1; swe; Apnh; Nhe1 | U51112 | transmembrane protein; growth factor activatable; similar to the Rattus norvegicus product encoded by GenBank Accession Number M85299; Mus musculus Na+/H+ exchanger (NHE-1) mRNA, complete cds.; solute carrier family 9 (sodium/hydrogen exchanger), member 1 |
| 162230_r_at | 2.46 | Rbbp9 | AV128327 | AV128327 Mus musculus C57BL/6J 11-day embryo Mus musculus cDNA clone 2700056L24, mRNA sequence. |
| 93524_i_at | 2.46 | Ube2j2 | AI839376 | UI-M-AN0-acj-h-05-0-UI.s1 NIH_BMAP_MBG Mus musculus cDNA clone UI-M-AN0-acj-h-05-0-UI 3', mRNA sequence.; ubiquitin-conjugating enzyme E2, J2 homolog (yeast) |
| 162324_at | 2.41 | Lor | AV083081 | AV083081 Mus musculus tongue C57BL/6J adult Mus musculus cDNA clone 2300010P06, mRNA sequence. |
| 98795_at | 2.41 | TRbeta2; Nr1a2; Thrb1; Thrb2; T3R[b]; T3Rbeta; c-erbAbeta | U15548 | Mus musculus beta 2 thyroid hormone receptor (TRbeta2) gene, exon 7 and complete cds. |
| 103003_i_at | 2.41 | Cd44; Cd44; Ly-24; Pgp-1; HERMES; AW121933; AW146109 | U57611 | 3' end of coding region for the major form of soluble CD44; hyaluronan receptor; Mus musculus cell surface glycoprotein CD44 (Cd44) mRNA, partial cds, exon 15 (v10). |
| 98598_at | 2.41 | C78891 | C78891 | C78891 Mouse 3.5-dpc blastocyst cDNA Mus musculus cDNA clone J0056H10 3' similar to Mouse MHC class II IE antigen beta chain (E-beta) gene, mRNA, mRNA sequence. |
| 99331_at | 2.41 | Apeg1 | AW125581 | UI-M-BH2.2-aqj-d-01-0-UI.s1 NIH_BMAP_M_S3.2 Mus musculus cDNA clone UI-M-BH2.2-aqj-d-01-0-UI 3', mRNA sequence.; aortic preferentially expressed gene 1 |
| 161607_r_at | 2.41 | Gjb6 | AV346646 | AV346646 RIKEN full-length enriched, adult male olfactory bulb Mus musculus cDNA clone 6430581O15 3', mRNA sequence. |
| 95949_at | 2.41 | DXErtd11e | C76382 | C76382 Mouse 3.5-dpc blastocyst cDNA Mus musculus cDNA clone J0009F07 3', mRNA sequence.; Mus musculus transcribed sequences |
| 162051_r_at | 2.41 | Svs6 | AV380774 | AV380774 RIKEN full-length enriched, adult male epididymis Mus musculus cDNA clone 9230101P15 3', mRNA sequence.; seminal vesicle secretion 6 |
| 161204_r_at | 2.41 | 8430421H08Rik | AV260414 | AV260414 RIKEN full-length enriched, adult male testis (DH10B) Mus musculus cDNA clone 4930413B17 3', mRNA sequence. |
| 103317_at | 2.41 | Coch-5B2; Coch; Coch-5B2; D12H14S564E | AF006741 | Mus musculus Coch-5B2 mRNA, complete cds.; coagulation factor C homolog (Limulus polyphemus) |
| 162419_r_at | 2.35 | 2610001J05Rik | AV323674 | AV323674 RIKEN full-length enriched, 11 days embryo head Mus musculus cDNA clone 6230411M08 3', mRNA sequence. |
| 97133_at | 2.35 | AA673488 | AA673488 | vp49f07.r1 Knowles Solter mouse 2 cell Mus musculus cDNA clone IMAGE:1080037 3', mRNA sequence.; Mus musculus 3 days neonate thymus cDNA, RIKEN full-length enriched library, clone:A630054K02 product:hypothetical protein, full insert sequence |
| 92612_at | 2.35 | vlacsr; Slc27a5; FATP5; FACVL3; VLCSH2; Vlacsr; VLCS-H2 | AJ223959 | strong expression in liver, low expression in lung, brain, testis, spleen and skeletal muscle, no expression in kidney and heart; Mus musculus mRNA for very-long-chain acyl-CoA synthetase related protein (VLACSR).; solute carrier family 27 (fatty acid transporter), member 5 |
| 100716_at | 2.35 | Has1; HAS | D82964 | Mus musculus mRNA for hyaluronan synthase, complete cds.; hyaluronan synthase1 |
| 161751_f_at | 2.35 | 1700020M16Rik | AV324916 | AV324916 RIKEN full-length enriched, 11 days embryo head Mus musculus cDNA clone 6230430A11 3', mRNA sequence. |
| 161958_at | 2.35 | 2610301D06Rik | AV221082 | AV221082 RIKEN full-length enriched, 14, 17 days embryo head Mus musculus cDNA clone 3230402N09 3' similar to M55409 Homo sapiens pancreatic tumor-related protein mRNA, mRNA sequence. |
| 99809_at | 2.30 | pax2; Pax2; Pax-2 | X55781 | M.musculus pax2 gene. |
| 161262_r_at | 2.30 | Odf2 | AV312779 | AV312779 RIKEN full-length enriched, adult male thymus Mus musculus cDNA clone 5830405I07 3', mRNA sequence. |
| 102393_at | 2.30 | Cryaa; Crya1; lop18; Acry-1; Crya-1; DAcry-1 | J00376 | alpha-A-crystallin; Mouse lens alpha-A-crystallin mRNA, 3' end.; crystallin, alpha A |
| 161310_at | 2.30 | Mef2d | AV079187 | AV079187 Mus musculus stomach C57BL/6J adult Mus musculus cDNA clone 2210408E11, mRNA sequence. |
| 162376_r_at | 2.30 | Ptprl | AV243403 | AV243403 RIKEN full-length enriched, 0 day neonate head Mus musculus cDNA clone 4831420F13 3' similar to U55057 Mus musculus receptor protein tyrosine phosphatase-lamda (ptp-lambda) mRNA, mRNA sequence. |
| 100725_at | 2.30 | Nqo1; Ox1; QR1; Dia4; NMO1; Ox-1; Nmo-1; Nmor1 | M36660 | NAD(P)H oxidoreductase (EC 1.6.99.2); Mouse NAD(P)H menadione oxidoreductase mRNA, complete cds.; NAD(P)H dehydrogenase, quinone 1 |
| 103499_at | 2.30 | Vwf | AI843063 | UI-M-AK1-aeq-h-10-0-UI.s1 NIH_BMAP_MHY_N Mus musculus cDNA clone UI-M-AK1-aeq-h-10-0-UI 3', mRNA sequence.; Von Willebrand factor homolog |
| 161639_f_at | 2.30 | Gast | AV062425 | AV062425 Mus musculus small intestine C57BL/6J adult Mus musculus cDNA clone 2010003E11, mRNA sequence.; gastrin |
| 93526_at | 2.30 | Stra13 | U95004 | Mus musculus D9 splice variant 2 mRNA, complete cds.; stimulated by retinoic acid 13 |
| 102261_f_at | 2.30 | Col13a1 | U30292 | Mus musculus collagen type XIII alpha-1 chain mRNA, complete cds.; procollagen, type XIII, alpha 1 |
| 92812_f_at | 2.30 | Defcr1 | U02995 | Mus musculus cryptdin-1 (Def) gene, exon 2 and complete cds. |
| 97235_f_at | 2.24 | Apobec2 | AW124988 | UI-M-BH2.1-apv-d-12-0-UI.s1 NIH_BMAP_M_S3.1 Mus musculus cDNA clone UI-M-BH2.1-apv-d-12-0-UI 3', mRNA sequence.; apolipoprotein B editing complex 2 |
| 95663_at | 2.24 | type IV collagenase; Mmp2; GelA; Clg4a; MMP-2 | M84324 | 72-kDa; Mus musculus type IV collagenase mRNA, complete cds.; matrix metalloproteinase 2 |
| 104417_at | 2.24 | Flt4 | AW048779 | UI-M-BH1-amg-b-01-0-UI.s1 NIH_BMAP_M_S2 Mus musculus cDNA clone UI-M-BH1-amg-b-01-0-UI 3', mRNA sequence.; FMS-like tyrosine kinase 4 |
| 101140_at | 2.24 | Htr1a; Gpcr18 | U39391 | 5-HT1A receptor; Plasma membrane 7-transmembrane receptor; Mus musculus serotonin1A receptor mRNA, complete cds.; 5-hydroxytryptamine (serotonin) receptor 1A |
| 101321_r_at | 2.24 |  | L28059 | Mus musculus Ig B cell antigen receptor gene, complete cds. |
| 102156_f_at | 2.24 | IgM | M80423 | putative; Mus castaneus IgK chain gene, C-region, 3' end. |
| 98803_at | 2.24 | kid-1; Zfp354a; kid1; Tcf17 | L77247 | putative; Mus musculus zinc finger protein (kid-1) gene, complete cds.; zinc finger protein 354A |
| 93893_f_at | 2.24 | Klra3; 5E6; Nk2; Nk-2; Ly49c; Nk2.1; NK-2.1 | U56404 | natural killer cell receptor; Mus musculus strain C57BL/6 natural killer cell receptor Ly-49C mRNA, complete cds.; killer cell lectin-like receptor, subfamily A, member 3 |
| 160527_at | 2.24 | mac25; Igfbp7; Fstl2; mac25 | AB012886 | Mus musculus mRNA for mac25, complete cds.; insulin-like growth factor binding protein 7 |
| 161733_at | 2.24 | Sqrdl | AV278406 | AV278406 RIKEN full-length enriched, adult male testis (DH10B) Mus musculus cDNA clone 4933401O22 3', mRNA sequence. |
| 96764_at | 2.24 | IIGP; AW111922; Iigp; IIGP1 | AJ007971 | Mus musculus mRNA for IIGP protein.; expressed sequence AW111922 |
| 92203_s_at | 2.24 | Cd6 | U37543 | Mus musculus T cell surface glycoprotein CD6 mRNA, complete cds.; CD6 antigen |
| 98480_s_at | 2.24 | Ren1; Rnr; Rn-1; Ren-1; Ren-A; Ren1d | M32352 | renin (Ren-1-d); Mouse renin (Ren-1-d) gene, complete cds. |
| 99152_at | 2.24 | Fmod; SLRR2E | X94998 | M.musculus mRNA for fibromodulin. |
| 93940_at | 2.24 | Pon3; Pon3; 2810004E20 | L76193 | Mus musculus paraoxonase-3 (Pon3) mRNA, complete cds.; paraoxonase 3 |
| 92687_at | 2.24 | Smarca4 | AA097203 | mm35h09.r1 Stratagene mouse skin (#937313) Mus musculus cDNA clone IMAGE:523553 5' similar to TR:G996018 G996018 BRG1 PROTEIN. ;, mRNA sequence.; SWI/SNF related, matrix associated, actin dependent regulator of chromatin, subfamily a, member 4 |
| 160671_at | 2.24 | Cln8 | AW124836 | UI-M-BH2.1-apk-e-10-0-UI.s1 NIH_BMAP_M_S3.1 Mus musculus cDNA clone UI-M-BH2.1-apk-e-10-0-UI 3', mRNA sequence.; ceroid-lipofuscinosis, neuronal 8 |
| 101660_at | 2.22 | Mtr; MS; AI894170; D830038K18Rik | AA415486 | vc52d03.r1 Knowles Solter mouse 2 cell Mus musculus cDNA clone IMAGE:778181 3' similar to WP:R03D7.1 CE01609 5-METHYLTETRAHYDROFOLATE-HOMOCYSTEINE METHYLTRANSFERASE ;, mRNA sequence.; Mus musculus 16 days neonate thymus cDNA, RIKEN full-length enriched library, clone:A130029B16 product:5-methyltetrahydrofolate-homocysteine methyltransferase, full insert sequence |
| 98856_at | 2.19 | Ptgdr; DP; PGD | D29765 | prostanoid DP receptor; Mus musculus gene for prostaglandin D receptor, complete cds and exon 2. |
| 103517_at | 2.19 | 4930565N07Rik | AA822898 | vp18g10.r1 Soares_mammary_gland_NbMMG Mus musculus cDNA clone IMAGE:1069026 5' similar to TR:Q13341 Q13341 LYSP100-A. ;, mRNA sequence. |
| 99498_at | 2.19 | Glns-ps1 | M60803 | intronless; putative; Mouse intronless glutamine synthetase gene, complete cds. |
| 93226_i_at | 2.19 | Igk-V8 | Z70661 | Artificial mRNA for single chain antibody scFv (scFvCPSQ). |
| 160523_at | 2.19 | Mpa2; Gbp4; Mag-2; Mpa-2 | M81128 | putative; putative; Mouse putative guanylate binding protein mRNA, complete cds.; macrophage activation 2 |
| 162038_f_at | 2.19 | A530057M15Rik | AV372745 | AV372745 RIKEN full-length enriched, adult male colon Mus musculus cDNA clone 9030612C19 3', mRNA sequence. |
| 101433_at | 2.19 | MR1E; Mr1; H2ls | AF010452 | MHC class I related gene 1 isoform E; MHC class I related protein 1 isoform E; Mus musculus MHC class I related protein 1 isoform E (MR1E) pseudogene mRNA, complete sequence.; major histocompatibility complex, class I-related |
| 101863_at | 2.19 |  | C78246 | C78246 Mouse 3.5-dpc blastocyst cDNA Mus musculus cDNA clone J0044H11 3' similar to Unannotatable data, mRNA sequence. |
| 97371_at | 2.19 | 3100004P22Rik | AW120599 | UI-M-BH2.3-anx-d-07-0-UI.s1 NIH_BMAP_M_S3.3 Mus musculus cDNA clone UI-M-BH2.3-anx-d-07-0-UI 3', mRNA sequence.; RIKEN cDNA 3100004P22 gene |
| 104064_at | 2.19 | Slc9a3r2 | AW045908 | UI-M-BH1-aku-c-01-0-UI.s1 NIH_BMAP_M_S2 Mus musculus cDNA clone UI-M-BH1-aku-c-01-0-UI 3', mRNA sequence. |
| 104024_at | 2.14 | CYP3A25; Cyp3a25 | Y11995 | M.musculus mRNA for cytochrome P450IIIA25.; cytochrome P450, family 3, subfamily a, polypeptide 25 |
| 161150_at | 2.14 | Tk1 | AV227393 | AV227393 RIKEN full-length enriched, 14 days embryo liver Mus musculus cDNA clone 4430402A13 3' similar to M19438 Mouse cytosolic thymidine kinase mRNA clone pMtk9, mRNA sequence. |
| 161917_i_at | 2.14 | Pnmt | AV380429 | AV380429 RIKEN full-length enriched, adult male epididymis Mus musculus cDNA clone 9230023G19 3' similar to U11694 Rattus norvegicus WKY and SHRSP phenylethanolamine N-methyltransferase (PNMT) gene, exons 2 and 3, and, mRNA sequence.; phenylethanolamine-N-methyltransferase |
| 160224_at | 2.14 | Slc35a4 | AW047009 | UI-M-BH1-alp-g-09-0-UI.s1 NIH_BMAP_M_S2 Mus musculus cDNA clone UI-M-BH1-alp-g-09-0-UI 3', mRNA sequence.; solute carrier family 35, member A4 |
| 102672_g_at | 2.14 | CREB | X67719 | Cluster Incl X67719:CAMP responsive element binding protein 1 /cds=(0,983) /gb=X67719 /gi=288883 /ug=Mm.1376 /len=984 |
| 162439_at | 2.14 | Pou2af1 | AV346238 | AV346238 RIKEN full-length enriched, adult male olfactory bulb Mus musculus cDNA clone 6430575J05 3' similar to Z54283 M.musculus mRNA for B-cell-specific coactivator BOB.1/OBF.1, mRNA sequence. |
| 160956_r_at | 2.14 | AA407526 | AA655804 | vs44f02.r1 Stratagene mouse Tcell 937311 Mus musculus cDNA clone IMAGE:1149147 5', mRNA sequence.; expressed sequence AA407526 |
| 94143_at | 2.14 | GFAP; Gfap | X02801 | Mouse gene for glial fibrillary acidic protein (GFAP). |
| 162495_r_at | 2.14 | Bet1 | AV149387 | AV149387 Mus musculus C57BL/6J 10-11 day embryo Mus musculus cDNA clone 2810487I08, mRNA sequence. |
| 93639_r_at | 2.14 | IgL; Igl-V1 | M94349 | putative; Mus musculus immunoglobulin lambda chain (IgL) mRNA, complete cds.; immunoglobulin lambda chain, variable 1 |
| 98932_at | 2.14 | 4833427B12Rik | AI854638 | UI-M-BH0-ake-f-10-0-UI.s1 NIH_BMAP_M_S1 Mus musculus cDNA clone UI-M-BH0-ake-f-10-0-UI 3', mRNA sequence.; RIKEN cDNA 4833427B12 gene |
| 162290_f_at | 2.14 |  | AV376843 | AV376843 RIKEN full-length enriched, adult male cecum Mus musculus cDNA clone 9130217J15 3', mRNA sequence. |
| 93847_at | 2.14 | Ptprd | AW120669 | UI-M-BH2.3-anz-h-07-0-UI.s1 NIH_BMAP_M_S3.3 Mus musculus cDNA clone UI-M-BH2.3-anz-h-07-0-UI 3', mRNA sequence.; protein tyrosine phosphatase, receptor type, D |
| 101742_at | 2.14 | LOC56544; V2R2 | AF053986 | Mus musculus tissue-type vomeronasal neurons putative pheromone receptor V2R2 mRNA, complete cds.; tissue-type vomeronasal neurons putative pheromone receptor V2R2 |
| 92283_s_at | 2.14 | Il4; IgG1; Il-4 | X03532 | unnamed protein product; put. IgG1 factor precursor; Mouse mRNA for IgG1 induction factor.; interleukin 4 |
| 94337_at | 2.14 | Gas2; Gas-2 | M21828 | growth-arrest-specific gas2 protein; Mouse growth-arrest-specific (gas2) protein mRNA, complete cds.; growth arrest specific 2 |
| 102075_at | 2.14 | MMCP-Y1; Mcpt-ps1; MMCP-(Y)1 | X78543 | no functional product; M.musculus MCP-Y1 mRNA, serine protease pseudogene. |
| 101920_at | 2.14 | Pole2 | AF036898 | Mus musculus DNA polymerase epsilon small subunit mRNA, partial cds.; polymerase (DNA directed), epsilon 2 (p59 subunit) |
| 100305_g_at | 2.14 | H2-M10.1 | AI449957 | mr82e08.x1 Stratagene mouse heart (#937316) Mus musculus cDNA clone IMAGE:603974 3' similar to SW:1B01_GORGO P30379 CLASS I HISTOCOMPATIBILITY ANTIGEN, GOGO-B0101 ALPHA CHAIN PRECURSOR. ;, mRNA sequence.; histocompatibility 2, M region locus 10.1 |
| 96413_at | 2.14 | Akap18; Akap7; AKAP15; Akap18; 6430401D08 | AF047716 | AKAP18; Mus musculus A-kinase anchoring protein (Akap18) mRNA, complete cds.; A kinase (PRKA) anchor protein 7 |
| 97730_at | 2.09 | ABCR; Abca4; RmP; Abcr; Abc10; D430003I15Rik | AF000149 | rim protein; RmP; Mus musculus ATP-binding cassette transporter (ABCR) mRNA, complete cds.; ATP-binding cassette, sub-family A (ABC1), member 4 |
| 92602_at | 2.09 | Tcap | AJ223855 | Mus musculus telethonin genomic sequence. |
| 95111_i_at | 2.09 | Ypel1 | AW122632 | UI-M-BH2.2-aol-c-11-0-UI.s1 NIH_BMAP_M_S3.2 Mus musculus cDNA clone UI-M-BH2.2-aol-c-11-0-UI 3', mRNA sequence.; yippee-like 1 (Drosophila) |
| 160528_at | 2.09 |  | Z47352 | Cluster Incl Z47352:Protamine 3 /cds=(0,323) /gb=Z47352 /gi=1360004 /ug=Mm.541 /len=324 /NOTE=replacement for probe set(s) 100545_at on MG-U74A |
| 99928_at | 2.09 | MAP1B; Mtap1b; LC1; MAP5; MAP1B; Mtap5; Mtap-5; A230055D22 | X51396 | Mus musculus mRNA for microtubule-associated protein 1B (MAP1B gene).; microtubule-associated protein 1 B |
| 93399_at | 2.09 | Rai2; 3f8 | X76652 | M.musculus mRNA for 3f8. |
| 98135_r_at | 2.09 | 2600013N14Rik | AI851008 | UI-M-BH0-ajv-c-01-0-UI.s1 NIH_BMAP_M_S1 Mus musculus cDNA clone UI-M-BH0-ajv-c-01-0-UI 3', mRNA sequence.; RIKEN cDNA 2600013N14 gene |
| 161662_f_at | 2.09 | Hcngp-pending | AV124076 | AV124076 Mus musculus C57BL/6J 11-day embryo Mus musculus cDNA clone 2700016D05, mRNA sequence. |
| 97098_at | 2.09 | 1300017K07Rik | AI838360 | UI-M-AI0-aak-a-10-0-UI.s2 NIH_BMAP_MBS Mus musculus cDNA clone UI-M-AI0-aak-a-10-0-UI 3', mRNA sequence.; RIKEN cDNA 1300017K07 gene |
| 161420_r_at | 2.09 | Dpagt1 | AV324170 | AV324170 RIKEN full-length enriched, 11 days embryo head Mus musculus cDNA clone 6230418O08 3' similar to X65603 M.musculus mRNA for GlcNAc-1-P transferase, mRNA sequence. |
| 101658_f_at | 2.09 | Q8/9d; H2-Q8; Qa8; Qa-2; Qa-8; H-2Q8; Ms10t; MMS10-T | D90146 | Mus musculus Q8/9d gene for Qa-2 cell surface antigen, complete cds. |
| 102205_at | 2.09 | Mafb; kr; Krml; Kreisler | L36434 | Mus Musculus basic domain/leucine zipper transcription factor mRNA, 3' end of cds.; v-maf musculoaponeurotic fibrosarcoma oncogene family, protein B (avian) |
| 102788_s_at | 2.09 | Rgs; Pitx2; Brx1; Ptx2; Rieg; Brx1a; Brx1b; Otlx2; Munc30; Pitx2a; Pitx2b; Pitx2c; solurshin; 9430085M16Rik | U70132 | bicoid-related homeodomain protein; murine homolog of Rieger syndrome; Mus musculus bicoid-related homeodomain protein solurshin (Rgs) mRNA, partial cds.; paired-like homeodomain transcription factor 2 |
| 162086_r_at | 2.09 | Trim30 | AV316033 | AV316033 RIKEN full-length enriched, adult male thymus Mus musculus cDNA clone 5830433P22 3' similar to J03776 Mouse down regulatory protein (rpt-1r) of interleukin 2 receptor mRNA, mRNA sequence. |
| 102053_at | 2.05 | Plscr2; Plscr1 | AF015790 | phospholipid flip/flop enzyme; Mus musculus phospholipid scramblase 2 mRNA, complete cds. |
| 162286_r_at | 2.05 | A430096B05Rik | AV373294 | AV373294 RIKEN full-length enriched, adult male colon Mus musculus cDNA clone 9030622L03 3', mRNA sequence. |
| 161676_at | 2.05 | 3300001M08Rik | AV169462 | AV169462 Mus musculus head C57BL/6J 13-day embryo Mus musculus cDNA clone 3110084P11, mRNA sequence. |
| 92286_g_at | 2.05 | Il4 | AA967539 | ua07e02.r1 Soares_thymus_2NbMT Mus musculus cDNA clone IMAGE:1346042 5' similar to gb:M25892 Mus musculus interleukin 4 (MOUSE);, mRNA sequence. |
| 92470_f_at | 2.05 | Vh186.2/Jh2; Igh-6 | AF065324 | Mus musculus clone X1AC1701 immunoglobulin heavy chain variable region (Vh186.2/Jh2) mRNA, partial cds.; immunoglobulin heavy chain 6 (heavy chain of IgM) |
| 103974_at | 2.05 | Tmprss2 | AI005782 | ua82g08.r1 Soares_mammary_gland_NbMMG Mus musculus cDNA clone IMAGE:1364030 5', mRNA sequence.; transmembrane protease, serine 2 |
| 92535_at | 2.05 | EBF; Ebf1; O/E-1; Olf-1 | L12147 | Mus musculus (clone 0EBF17) early B-cell factor (EBF) mRNA, complete cds.; early B-cell factor 1 |
| 92433_at | 2.05 | Kif5c; Kif5c; KINN; NKHC; NKHC2; NKHC-2 | AF067180 | Mus musculus kinesin heavy chain (Kif5c) mRNA, complete cds.; kinesin family member 5C |
| 98843_at | 2.05 | Zic2 | D70848 | Mus musculus mRNA for Zic2 protein, complete cds.; zinc finger protein of the cerebellum 2 |
| 100760_at | 2.05 | Dsc1 | X97986 | Cluster Incl X97986:M.musculus mRNA for desmocollin type 1 /cds=(151,2649) /gb=X97986 /gi=1707586 /ug=Mm.909 /len=2987 |
| 161152_r_at | 2.05 | Ubce7ip3-pending | AV230260 | AV230260 RIKEN full-length enriched, 0 day neonate skin Mus musculus cDNA clone 4631427E13 3' similar to AB011369 Rattus norvegicus mRNA for RBCK2, mRNA sequence. |
| 92689_at | 2.05 | Il18bp; MC54L; Igifbp; IL-18BP | AB019505 | Mus musculus mRNA for interleukin-18 binding protein, complete cds.; interleukin 18 binding protein |
| 92355_at | 2.05 | Fog-2; Zfpm2; FOG2; FOG-2; B330005D23Rik | AF107306 | Mus musculus zinc-finger protein FOG-2 (Fog-2) mRNA, complete cds.; zinc finger protein, multitype 2 |
| 100556_at | 2.05 | 2310046H11Rik | AW121930 | UI-M-BH2.3-aoj-d-12-0-UI.s1 NIH_BMAP_M_S3.3 Mus musculus cDNA clone UI-M-BH2.3-aoj-d-12-0-UI 3', mRNA sequence.; RIKEN cDNA 2310046H11 gene |
| 104073_at | 2.05 | Cugbp1 | AI847630 | UI-M-AP1-agg-e-11-0-UI.s1 NIH_BMAP_MST_N Mus musculus cDNA clone UI-M-AP1-agg-e-11-0-UI 3', mRNA sequence.; CUG triplet repeat, RNA binding protein 1 |
| 98848_at | 2.05 | Sh3d4; SH3P3 | U58889 | contains SH3 domain; Mus musculus SH3-containing protein SH3P3 mRNA, partial cds.; SH3 domain protein 4 |
| 161343_r_at | 2.00 | 9030616F16 | AV136210 | AV136210 Mus musculus C57BL/6J 10-11 day embryo Mus musculus cDNA clone 2810025F01, mRNA sequence. |
| 101351_at | 2.00 | Casr-rs2; Gprc2a-rs2; Casr-rs2 | AF022251 | putative calcium-sensing receptor homolog; Mus musculus calcium-sensing receptor related protein 2 (Casr-rs2) gene, partial cds. |
| 160910_at | 2.00 | Ghrh; Ghrf | M31654 | growth hormone-releasing hormone precursor; Mouse growth hormone-releasing hormone mRNA, complete cds.; growth hormone releasing hormone |
| 161312_r_at | 2.00 | Sprr1a | AV085264 | AV085264 Mus musculus tongue C57BL/6J adult Mus musculus cDNA clone 2310010O13, mRNA sequence. |
| 96603_at | 2.00 | Qscn6 | AW123556 | UI-M-BH2.1-aqb-g-04-0-UI.s1 NIH_BMAP_M_S3.1 Mus musculus cDNA clone UI-M-BH2.1-aqb-g-04-0-UI 3', mRNA sequence.; quiescin Q6 |
| 97287_at | 2.00 | 4933412D19Rik | AW046638 | UI-M-BH1-ald-d-09-0-UI.s1 NIH_BMAP_M_S2 Mus musculus cDNA clone UI-M-BH1-ald-d-09-0-UI 3', mRNA sequence.; Mus musculus transcribed sequence with weak similarity to protein ref:NP_002605.1 (H.sapiens) PDZ domain containing 1 [Homo sapiens] |
| 101353_at | 2.00 | Casr-rs3; Gprc2a-rs3; Casr-rs3 | AF022252 | putative calcium-sensing receptor homolog; Mus musculus calcium-sensing receptor related protein 3 (Casr-rs3) gene, partial cds. |
| 95771_i_at | 2.00 | frizzled 4; Fzd4; Fz4 | U43317 | putative transmembrane receptor; Mus musculus putative transmembrane receptor (frizzled 4) mRNA, complete cds.; frizzled homolog 4 (Drosophila) |
| 97162_at | 2.00 |  | AI451676 | mu58d10.x1 Soares mouse lymph node NbMLN Mus musculus cDNA clone IMAGE:643603 3', mRNA sequence.; Mus musculus transcribed sequences |
| 161232_r_at | 2.00 | Gpiap1 | AV276689 | AV276689 RIKEN full-length enriched, adult male testis (DH10B) Mus musculus cDNA clone 4932435P03 3' similar to U18773 Mus musculus GPI-anchored protein p137 mRNA, mRNA sequence. |
| 99605_at | 2.00 | 1810015H18Rik | AA536968 | vj86e02.r1 Knowles Solter mouse 2 cell Mus musculus cDNA clone IMAGE:943994 3', mRNA sequence. |
| 100300_at | 2.00 | Cybb; Cybb; Cgd; Nox2; gp91phox; gp91<phox> | U43384 | heme binding membrane glycoprotein, also flavin and NADPH binding domains. gp91phox is associated with a 22 kD peptide (p22phox) to form cytochrome b558, the redox carrier in the phagocyte NADPH oxidase that generates superoxide; murine homolog of human CYBB, site of mutations in X-linked granulomatous disease; subunit of b-type cytochrome heterodimer; Mus musculus gp91phox (Cybb) mRNA, complete cds.; cytochrome b-245, beta polypeptide |
| 161274_at | 2.00 | Sqle | AV324302 | AV324302 RIKEN full-length enriched, 11 days embryo head Mus musculus cDNA clone 6230421L08 3' similar to D42048 Mouse mRNA for squalene epoxidase, mRNA sequence. |
